# Synthesis and conformational preferences of short analogues of antifreeze glycopeptides (AFGP)

**DOI:** 10.3762/bjoc.15.162

**Published:** 2019-07-16

**Authors:** Małgorzata Urbańczyk, Michał Jewgiński, Joanna Krzciuk-Gula, Jerzy Góra, Rafał Latajka, Norbert Sewald

**Affiliations:** 1 Department of Bioorganic Chemistry, Faculty of Chemistry, Wroclaw University of Science and Technology, Wybrzeże Wyspianskiego 27, Wroclaw, PL-50-370, Poland; 2 Organic and Bioorganic Chemistry, Department of Chemistry, Bielefeld University, Universitätsstraße 25, Bielefeld, D-33615, Germany

**Keywords:** antifreeze glycopeptides, conformational preferences, NMR, PP II, solid phase synthesis

## Abstract

Antifreeze glycoproteins are a class of biological agents which enable living at temperatures below the freezing point of the body fluids. Antifreeze glycopeptides usually consist of repeating tripeptide unit (-Ala-Ala-Thr*-), glycosylated at the threonine side chain. However, on the microscopic level, the mechanism of action of these compounds remains unclear. As previous research has shown, antifreeze activity of antifreeze glycopeptides strongly relies on the overall conformation of the molecule as well an on the stereochemistry of amino acid residues. The desired monoglycosylated analogues with acetylated amino termini and the carboxy termini in form of *N*-methylamide have been synthesized. Conformational nuclear magnetic resonance (NMR) studies of the designed analogues have shown a strong influence of the stereochemistry of amino acid residues on the peptide chain stability, which could be connected to the antifreeze activity of these compounds. A better understanding of the mechanism of action of antifreeze glycopeptides would allow applying these materials, e.g., in food industry and biomedicine.

## Introduction

Certain species of polar and sub-polar fish created evolutionary strategies to survive in water at temperatures below the colligative freezing point (−1.8 °C). Antifreeze glycoproteins and antifreeze glycopeptides (AFGPs) are one group of biological antifreeze agents that prevent ice crystal growth in body fluids of certain ectotherm species [1–3]. These macromolecules are composed of 4 to 55 repeating tripeptide units (-Ala-Ala-Thr*-), where each hydroxy group in the threonine side chain is glycosylated with the disaccharide β-ᴅ-galactosyl-(1→3)-α-*N*-acetyl-ᴅ-galactosamine [4].

Even though the macroscopic properties of AFGPs, such as thermal hysteresis [5–6] and ice recrystallization inhibition [7–9] have been well described, the microscopic mechanism of ice–glycoprotein interaction is still unknown. Therefore, it is an interesting area for further research [3,10–11]. Some studies have highlighted the role of irreversible adsorption of AFGPs onto growing ice surface and incorporation of hydroxy groups of the disaccharide subunit into the ice lattice [12], others investigated the adsorption process on different surfaces via atomic force microscopy [10,13–14]. The antifreeze activity has been correlated with long-range perturbation of hydration dynamics [15]. Latest molecular dynamics simulations suggest that AFGP reversibly binds onto the ice surface via hydrophobic groups and “walks” on the flat ice surface, which is a completely new hypothesis [16].

The antifreeze activity of these biopolymers is highly related to the stereochemical features of the peptide backbone and the glycosyl moiety. Previously it was assumed that the saccharide moiety is conformationally well-defined by the peptide backbone and the whole structure adopts a polyproline II (PP II) helix [17]. Antifreeze activity strongly relies on the stereochemistry of the amino acid residues. Earlier studies have shown that AFGP analogues containing either an 2-acetamido-2-deoxy-ᴅ-galactopyranosyl residue (ᴅ-GalNAc) attached to an *allo*-threonine-containing PP II helical peptide or containing ᴅ-GalNAc attached to a fully ᴅ-configured PP II helical peptide (retro-inverso) are inactive, while the corresponding ʟ-configured peptide showed antifreeze activity [18]. The synthesis of the retro-inverso analogue had been envisaged to prove that changing the amino acid configuration from ʟ to ᴅ and reversing of the peptide sequence should place the side chains in a similar manner as for the parent compound. Interestingly, replacing alanine by proline in AFGP analogues, which occasionally occurs in smaller natural AFGP (and furthermore proline is a precursor of the PP II helix) significantly decreased the inhibitory effect on the ice recrystallization of the compounds [19].

Here we present the synthesis of a series of model peptides comprising structural motifs from antifreeze glycopeptides. The target compounds were designed on the basis of a literature review and contained a ᴅ-GalNAc subunit attached to an ʟ-Thr or ᴅ-Thr residue, respectively. The amino termini of the glycopeptides were acetylated, and the carboxy terminus was present as the *N*-methylamide with the aim of excluding pH-dependent charge effects and to simulate a protein environment. The introduction of a methyl amide function at the C-terminus of glycopeptides was carried out on solid phase. The *N*-methylation of the peptide terminus on solid support was an efficient four-step procedure. This innovative synthetic approach enabled easy modification without purification step, which is necessary after reaction in solution. Besides, commonly existing solution-phase methods involve harsh reaction conditions [20–21].

We performed a study to further determine structural motifs required for antifreeze activity, as well as to clarify the influence of hydrogen bonds on the preferred conformation of the molecule. Model AFGP analogues were investigated by NMR in order to elucidate the stereochemical influence of the amino acid configuration on the three-dimensional structure of the glycopeptides.

A better understanding of the correlation between structure and activity of AFGPs would not only improve the basic knowledge on molecular recognition, but could also help designing new tailored antifreeze agents for many applications, e.g., cooling systems, coating technology, ice-templating, food industry, agriculture, biomedical cryopreservation [22–24].

## Results and Discussion

### Peptide synthesis

**Figure 1:**
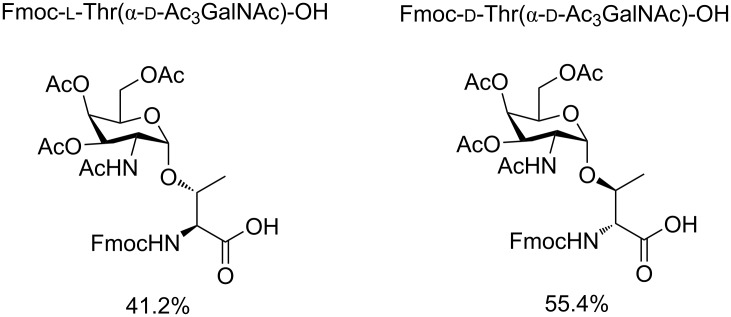
Glycosylated building blocks prepared for solid phase peptide synthesis (SPPS).

Starting from ᴅ-(+)-galactose, we obtained the 3,4,6-tri-*O*-acetyl-ᴅ-galactal in an excellent yield and transformed it into 3,4,6-tri-*O*-acetyl-2-azido-2-desoxy-α-ᴅ-galactopyranosyl chloride by one pot azidochlorination [25]. Glycosylated building blocks, containing ʟ- or ᴅ-Thr were synthesized (Figure 1) over three steps [26].

**Scheme 1:**
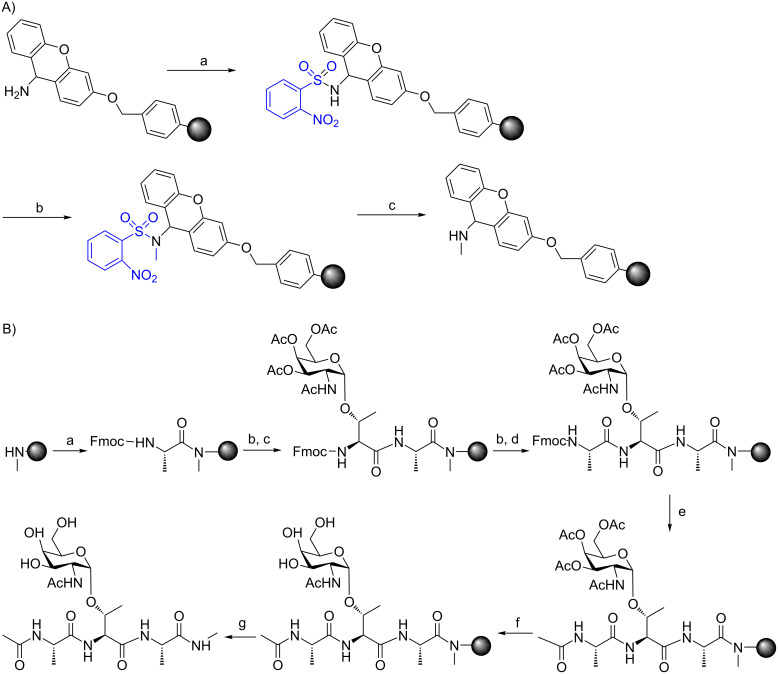
A) Modification of Fmoc-Sieber-PS resin: a. piperidine in DMF (20% v/v), rt; 3 × 10 min; b. *o-*NBS-Cl (4 equiv), collidine (10 equiv) NMP, rt, 2 h, twice; c. triphenylphosphine (PPh_3_, 5 equiv), methanol (MeOH, 10 equiv), DIAD (5 equiv), THF, rt, 2 h, twice; d. HSCH_2_CH_2_OH (10 equiv), DBU (5 equiv), NMP, rt, 2 h, twice; B) SPPS of model AFGP analogues 1 and 2: a. Fmoc-Ala-OH∙H_2_O (4 equiv), HOAt (4 equiv), HATU (4 equiv), DIEA (8 equiv), NMP, rt, 2 h; b. piperidine 20% v/v in DMF 3 × 10 min; c. Fmoc-L-Thr(α-GalNAc)-OH (1 equiv), HOAt (2.25 equiv), HATU (2.8 equiv), DIEA (2.75 equiv), DMF, rt, 3 h; d. Fmoc-L-Ala-OH∙H_2_O (4 equiv), TBTU (4 equiv), DIEA (4 equiv), DMF, rt, 2 h; e. Ac_2_O (50 equiv), DIEA (12.5 equiv), HOBt (1 equiv), DMF, rt, 40 min; f. 1.0 M hydrazine in THF, rt, 2 h; g. 7–10% TFA in DCM, rt, cleavage 10 × 5 min.

The glycosylated building blocks were used in SPPS of model tri- and pentapeptides. The N-terminal end of the products was acetylated and the carboxy terminus was in a form of *N*-methylamide. The introduction of a methylamide function at the C-terminal end was performed on solid phase. In order to achieve this, the Fmoc-Sieber-PS resin was modified (Scheme 1A). This type of resin enabled cleavage of peptides under mild conditions, hence without destroying fragile *O*-glycosidic bond. Moreover, performing this reaction on the resin has eliminated the need for product purification after each step.

Modification of the resin was performed according to the protocol published by J. Chatterjee and co-workers [27]. This practical and straightforward strategy involved direct *N*-methylation of the resin linker in an efficient four-step procedure compatible with Fmoc-based SPPS.

In the first step the Fmoc group was cleaved by treatment with 20% piperidine in DMF giving the free amino function on the resin linker. Then the resin was washed with DCM, DMF and NMP. In the next step the free amino groups of the resin were *o-*NBS protected by treatment of 4-nitrobenzenesulfonyl chloride (*o-*NBS-Cl) and *sym*-collidine in NMP (Scheme 1A, b).

Subsequently the resin was *N*-methylated by Mitsunobu reaction (Scheme 1A, c). Triphenylphosphine (PPh_3_) was dissolved in dry THF and methanol and shaken with the resin. After two minutes the diisopropyl azodicarboxylate (DIAD) solution was added dropwise to the reaction mixture. Once the reaction was complete, the *o-*NBS protecting group was removed by using 2-mercaptoethanol and 1,8-diazabicyclo[5.4.0]undec-7-ene (DBU). The Kaiser test did not reveal any presence of free NH_2_ groups, which proved formation of the secondary amine.

Afterwards, the first amino acid was attached to the modified resin. Coupling of alanine to the secondary amine was challenging. Therefore, the reaction time was extended to two hours, using the efficient coupling reagent HATU, combined with HOAt. Loading of the resin was determined spectrophotometrically by measuring the absorption of the piperidine-dibenzofulvene adduct formed during Fmoc deprotection (20% v/v piperidine in DMF) at wavelength 290 nm. The obtained loadings were ranging from 0.36–0.60 mmol/g, while the original loading was 0.61 mmol/g. Lower loading of the resin was used in order to enable quantitative incorporation of the sterically hindered, glycosylated building blocks.

The synthesis of tri- and pentapeptides (Scheme 1B) was performed manually on solid phase [28]. The obtained glycopeptides were cleaved from the resin by treatment with 7–10% TFA in DCM. The cleavage cocktail was co-evaporated with toluene with the purpose of avoiding high TFA concentrations. The crude products were precipitated in cold diethyl ether, if possible. Subsequently, the peptides were lyophilised in a mixture of water and acetonitrile (80:20 v/v) and purified by preparative RP-HPLC (see Supporting Information File 1).

**Figure 2:**
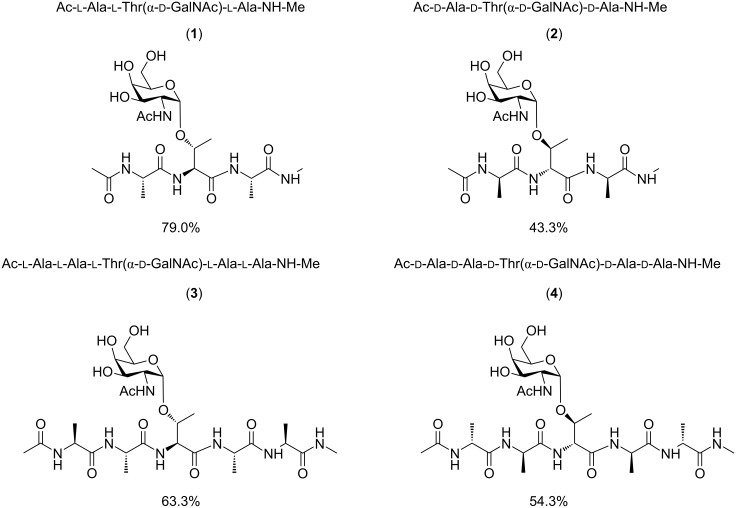
Model AFGP analogues.

The glycosylated threonine building blocks were successfully prepared using a previously published synthetic route. The modification of the PS-Sieber resin via the presented protocol turned out to be efficient and easy to control. Desired loadings of the resin were achieved by changing the reaction parameters (e.g., reaction time, amino acid equivalent). The synthesis of model tri- and pentapeptides containing ᴅ-GalNAc attached to ʟ- and ᴅ-threonine was performed as outlined in Figure 2. During this part of synthesis, the best yields were achieved for incorporation of natural amino acids into the peptide chain, where 1.2 equiv of glycosylated threonine were applied. NMR measurements were performed in order to elucidate the stereochemical influence of the amino acid configuration on the three-dimensional structure of antifreeze glycopeptides.

### NMR spectroscopy and structural calculation

Based on 1D and 2D correlation spectroscopy experiments, including H,H-COSY, H,H-TOCSY, H,C-HSQC and H,C-HMBC spectra, assignment of the resonances of the studied peptides was performed. Designing and obtaining short, simplified AFGP analogues avoided overlapping of signals.

#### Temperature coefficient of the amide protons

The temperature coefficient factor of amide protons dδ/dT [ppb/K] is one of the parameters, which can be used to determine the existence of hydrogen bonds in peptide chains. The amide protons involved in the formation of an intramolecular hydrogen bond are characterized by chemical shifts that are independent on the temperature. When the amide proton is involved in a hydrogen bond, its temperature factor value is lower than 4.0 ppb/K [29–30]. Values of the measured temperature coefficient factors (see Table 1) suggest that the conformation of each of the investigated pentapeptides is stabilized by one hydrogen bond, created by the amide proton of Ala^4^. In the case of peptide **4** we could observe the strongest hydrogen bond (Table 1). Previous studies pointed out the role of specific hydrogen bonds between the peptide backbone and GalNAc moiety, which are responsible for creating the extended conformation [31–32]. However, further NMR investigations in this area found no evidence of an intramolecular hydrogen bond, or the existence of a very weak interaction, between the carbonyl group of the serine residue and the amide proton of GalNAc unit [33]. This correlates with our results obtained for longer peptides containing the same sugar moiety attached to threonine. Moreover, Corzana et al. [33] highlighted the importance of surrounding solvent molecules, which are responsible for the existence of water pockets/bridges between the monosaccharide residue and peptide backbone, hence maintaining the defined conformation.

**Table 1:** Intramolecular hydrogen bonds – temperature coefficient -dδ/dT [ppb/K] in DMSO.

Amide proton	Glycopeptide	

	**3**	**4**

Ala^1^	5.6	5.1
Ala^2^	4.3	5.1
Thr^3^	4.8	4.8
GalNAc	5.2	5.8
Ala^4^	**3.5**	**1.4**
Ala^5^	7.6	8.4
CNMe^a^	6.4	6.3

^a^CNMe–HN of C-terminal group.

#### Structural investigation of the glycopeptides

Although the temperature factor gives important structural information, the most valuable and crucial data were obtained from the NOESY/ROESY spectra. Based on the intensity of the correlation peaks, proton–proton distances were calculated. The most important structural information was gained from the nontrivial inter-residual correlation signals. These experimental data were essential for the calculation of the conformational preferences of the examined glycopeptides. Based on the NOESY/ROESY experiments we obtained 6, 27, 10 and 50 distance constrains for peptide **1**, **2**, **3**, and **4**, respectively (see Supporting Information File 1). For each of the investigated peptides optimization of 1 000 stable conformers have been calculated with the usage of the XPLOR-NIH program [34]. The obtained conformations were clustered based on the main chain backbone dihedral angles (φ and ψ) using the AmberTools package [35].

**Figure 3:**
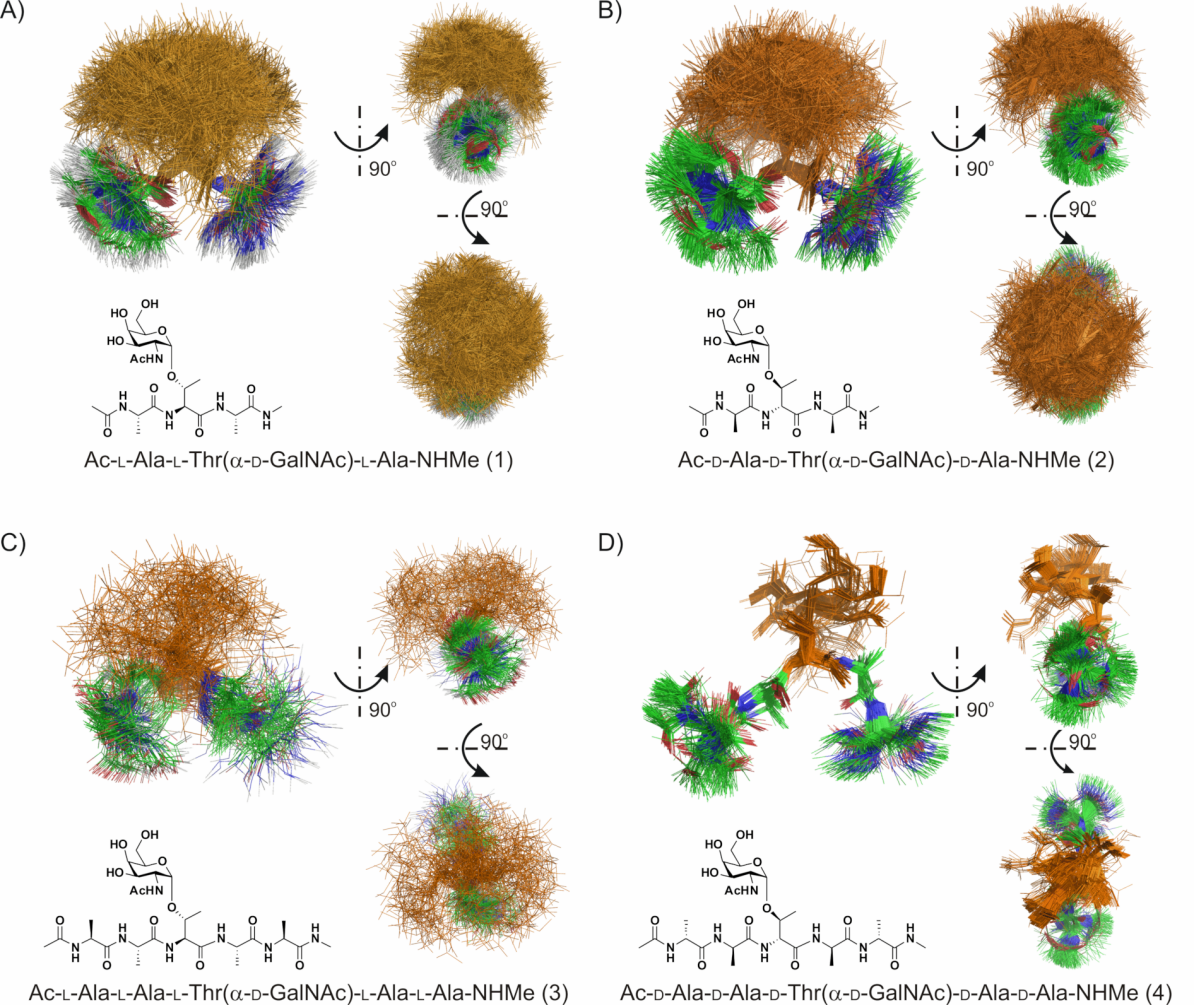
Conformational preferences of investigated glycopeptides.

The first analysis of the number of obtained distance constrains clearly suggested that peptides containing ʟ-amino acids should be much more flexible than analogues containing ᴅ-residues. Such observation is independent on the length of the peptide. In case of both tripeptides the performed calculations do not show any conformational preferences. Even regarding peptide **2**, for which 27 interatomic distances have been determined, no predominant conformer was found. As shown in Figure 3A and B both tripeptides indicate a fully flexible conformation. Corzana et al. suggested for the C- and N-protected, glycosylated ʟ-serine residue an extended conformation without intramolecular hydrogen bond between the amide proton of GalNAc and a carbonyl oxygen in the peptide main chain [33]. These conclusions had been based on the intensity of the observed NOE signals between main chain amide protons and α-protons. They conclude that the presence of strong NOE signals between H^α^*_i_* and H^N^*_i_*_+I_ protons (d_Hα_*_i_*_−HN_*_i_*_+1_) together with the weak one between H^N^*_i_* and H^N^*_i_*_+I_ protons (d_HN_*_i_*_-HN_*_i_*_+1_) or similar intensity of NOE signals between H^α^*_i_* and H^N^_i_ (d_Hα_*_−_*_HN1_) as well as H^N^_i+I_ (d_Hα_*_i_*_−HN_*_i_*_+1_) is a sufficient evidence for the presence of the extended structure. However, a similar analysis of the determined NOE interactions did not provide enough evidence to draw the same conclusions. Only the C-terminal part of **2** seems to adopt an extended conformation, as only for Ala^3^ the intensity of the d_Hα_*_i_*_−HN_*_i_* is comparable to the intensity of d_Hα_*_i_*_−HN_*_i_*_+1_ (see Supporting Information File 1, Table S4).

Regarding pentapeptide **3**, containing only ʟ-amino acid residues, we could define only one relatively stable conformation cluster (Table 2). However, this cluster was determined by dihedral angles only for three residues from Ala^1^ to Thr^3^ (Table 3). As shown in Table 2, all attempts to use more torsion angles did not allow defining a stable conformation covering the entire main chain. The observed unordered conformation is in agreement with the previously reported results, which suggested that one repeating unit comprising the Ala-Thr(GalNAc)-Ala motif is not enough to provide interresidual interaction, required for stabilization of the ordered conformation [36–37].

**Table 2:** Influence of used backbone dihedral onto cluster size of peptide **3**.

Used dihedrals	Size of the biggest cluster [%]

Ala^1^-Ala^5^	1.1
Ala^1^-Ala^4^	3.7
Ala^1^-Thr^3^	15.5
Ala^2^-Ala^5^	1.1
Ala^2^-Ala^4^	3.7
Thr^3^-Ala^5^	1.7

**Table 3:** Characterization of conformational clusters obtained for clustering using backbone dihedrals for Ala^1^-Thr^3^ of peptide **3** and **4**.

Cmpd	Size [%]	Ala^1^		Ala^1^		Thr^3^			Ala^4^		Ala^5^	
		φ	ψ	φ	ψ	φ	ψ	χ1	φ	ψ	φ	ψ

**3**	15.5	161.7 ± 58.8	3.2 ± 15.9	121.4 ± 55.5	19.2 ± 28.3	−49.8 ± 112.7	3.5 ± 55.5	52.3 ± 109.7	−57.9 ± 69.1	42.3 ± 99.5	−53.4 ±70.3	40.6 ± 98.9
**4**	64.5	63.8 ± 69.1	−114.3 ± 13.9	50.3 ± 86.9	−150.5 ± 7.9	88.1 ± 8.0	60.8 ± 13.7	−38.8 ± 15.4	60.3 ± 25.3	−120.3 ± 18.0	53.6 ± 78.1	−69.8 ± 86.6

In case of pentapeptide **4** the calculation resulted in 1 000 conformers. Cluster analysis based on all backbone dihedral angles allowed to identify multiclusters, containing approximately 1% of structures each. However, the clustering based only on the part of the main chain led to higher populated clusters. The biggest cluster has been found for clustering based on the φ, ψ torsion angles of Ala^2^-Thr^3^-Ala^4^. The biggest cluster contained more than 64% of all structures (see Table 4). Therefore, such observation can suggest high stability of the central part of the investigated glycopeptide, which made peptide **4** much more ordered than peptide **3**. Although peptide **4** adopted a highly stable conformation, at least in the central part, analysis of the main chain dihedral angles did not allow assigning any of the standard secondary structures. Analysis of the determined φ and ψ torsion angles (see Table 3) allowed only to conclude that peptide **4** did not adopt the often observed polyproline type helix [38] nor the extended conformation postulated by Corzana et al. [33].

**Table 4:** Influence of used backbone dihedral onto cluster size of peptide **4**.

Used dihedrals	Size of the biggest cluster [%]

Ala^1^-Ala^5^	1.1
Ala^1^-Ala^4^	5.7
Ala^1^-Thr^3^	11.1
Ala^2^-Ala^5^	3.9
Ala^2^-Ala^4^	64.5
Thr^3^-Ala^5^	7.8

**Figure 4:**
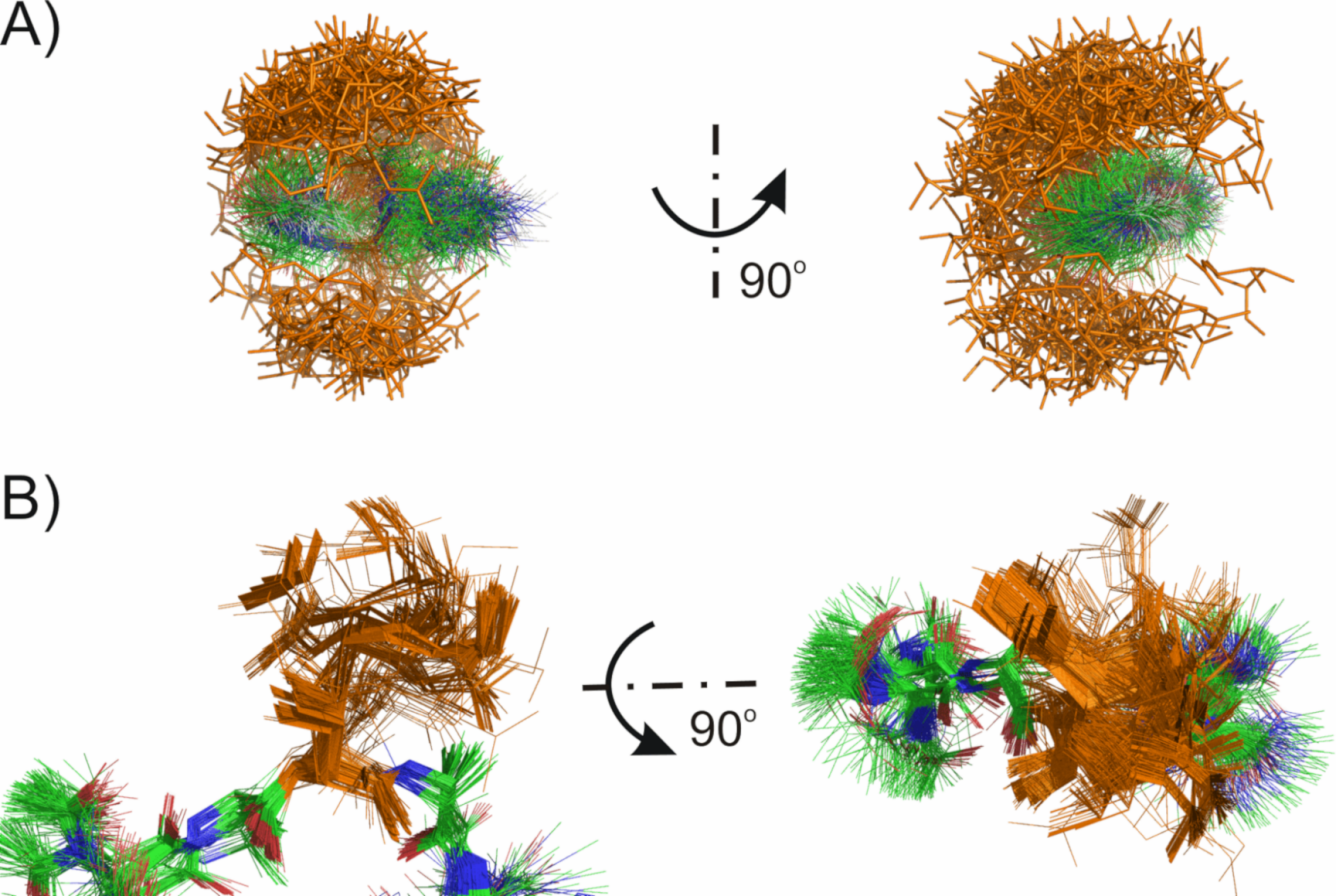
Conformational preferences of monosaccharide moiety. A) cluster 1 for glycopeptide **3**, B) cluster 1 for glycopeptide **4**.

Interestingly, also the arrangement of the monosaccharide moiety is much more ordered in glycopeptide **4** in comparison to peptide **3** (see Table 3 and Figure 4). Such situation is the result of numerous well defined intra- and interresidual NOE signals of sugar moiety protons (see Table S6, Supporting Information File 1). Although the orientation of the galactose ring is highly ordered, as shown the value of the temperature coefficient (see Table 1), its arrangement is not stabilized by an intramolecular hydrogen bond formed by the GalNAc amide proton. Instead, as revealed by the results of the structural calculation, the orientation of the galactose ring probably is locked due to interaction of the Ala^4^ amide proton and the side chain oxygen of Thr^3^. In case of peptide **3**, due to weaker intramolecular hydrogen bonds combined with a high flexibility of the peptide main chain, the fixed orientation of the galactose ring is not observed (see Figure 3 and Figure 4). Such conclusion seems to be confirmed by the small value of the temperature coefficient for this amide proton (see Table 1). The value found suggests that the formed hydrogen bond is strong and stable, much stronger than the one found for peptide **3**. This could explain the lacking antifreeze activity of the fully ᴅ-configured AFGP analogue. High flexibility of AFGP molecules, even in the presence of ice, was described as essential for their antifreeze activity [13–14,39]. In addition the high flexibility of these molecules enables the exposing of ice-binding groups onto the growing ice surface, hence constructing the amphipathic character of AFGPs, where the saccharide subunits are located on the same side of the molecule, constructing a hydrophilic face, while Ala–CH_3_ groups are forming a hydrophobic face [17].

## Conclusion

Our research, although does not fully explain the mechanism of action of AFGPs, provides interesting structural and conformational information about the influence of the amino acid stereochemistry on the peptide backbone stability. Designing short sequences of AFGP analogues allowed complete assignment and full interpretation of the NMR spectra.

The antifreeze activity of AFGP analogues is abolished by changing the stereochemistry on C^α^ and C^β^ carbons of the glycosylated Thr residue. The all-ᴅ-configured pentapeptide **4** turned out to adopt a much more rigid structure in comparison to the corresponding peptide **3** containing only ʟ-amino acids. Moreover, pentapeptide **4** showed the ability to “lock” the orientation of the carbohydrate ring. We assume that ʟ-configured AFGP analogues may exhibit antifreeze activity due to their higher flexibility, hence they can expose more ice-binding groups onto the ice surface, therefore, the amphipathic character of AFGPs might play a crucial role in their activity.

## Experimental

### Nuclear magnetic resonance

NMR spectra were recorded on a Bruker AMX600 spectrometer in deuterated dimethyl sulfoxide (DMSO-*d*_6_) at 298 K. Chemical shifts are given in ppm, relative to residual solvent signals (δ_H_ = 2.49 ppm, δ_C_ = 39.0 ppm). NMR spectral signal assignment and integration were carried out with Bruker TopSpin and SPARKY software [40]. For 2D ROESY, if it was possible, the separation between two geminal protons in −CH_2_− group was used as a reference in distance calculations, otherwise interatomic distances were calculated from the intensity of the cross and diagonal peaks. In both cases calculated distances were offset-corrected [41]. The temperature coefficient factors of amide protons were determined at a temperature ranging from 293 to 308 K. Conformational calculations for the investigated tripeptides **1** and **2** as well as pentapeptides **3** and **4** were performed with the X-PLOR NIH 3.5 program package [34].

### General methods

All air- and moisture-sensitive reactions were carried out in a dry argon atmosphere in flame-dried glass flasks. Dichloromethane (DCM) was freshly distilled from CaH_2_ and toluene from Na. *N,N*-Dimethylformamide (DMF) was distilled from ninhydrine. All amino acids, coupling reagents and the Fmoc-Sieber PS (*N*-(9*H*-fluoren-9-yl)methoxycarbonyl – Fmoc) resin were purchased from Iris Biotech (Marktredwitz, Germany). Other chemicals were bought from Sigma-Aldrich (Hamburg, Germany), Acros (Geel, Belgium), and VWR (Darmstadt, Germany). All chemicals were used as purchased, if not stated otherwise.

Analytical reversed-phase high-performance liquid chromatography (RP-HPLC) was carried out on a Thermo Separation Products system consisting of a UV 6000 diode array detector and a P 4000 pump equipped with a Phenomenex HPLC guard cartridge system (C12; 4 × 3.00 mm) and a Phenomenex Jupiter 4 µ Proteo 90 Å column (C12; 250 × 4.60 mm). Flow rate 1 mL min^−1^. Eluent A: H_2_O/CH_3_CN/TFA (trifluoroacetic acid – TFA) (95:4.9:0.1), eluent B: CH_3_CN/H_2_O/TFA (95:4.9:0.1). Preparative RP-HPLC was carried out with a Thermo Separation Products system consisting of a UV-1000 detector and a P-4000 pump equipped with a Vydac high-performance guard column (C18) and a Phenomenex Jupiter 10 µ Proteo 90 Å column (C12; 250 × 21.20 mm) or with a Hitachi MERCK LaChrom system consisting of a UV–vis L-7420 detector and a L7150 pump equipped with a Vydac high-performance guard column (C18) and a Phenomenex Jupiter 10 µ 300 Å column (C18; 250 × 21.20 mm). Flow rate 7.5 mL min^−1^. Eluent A: H_2_O/CH_3_CN/TFA (95:4.9:0.1), eluent B: CH_3_CN/H_2_O/TFA (95:4.9:0.1)

### Synthesis

The synthesis of glycosylated threonine building blocks containing ʟ- or ᴅ-Thr residues was performed according to a previously published route [26].

The Fmoc-Sieber-PS resin was *N*-methylated. Firstly, the Fmoc group was cleaved with 20% piperidine in DMF from the resin linker (3 × 10 min shaking). After washing the resin three times with DCM, DMF and 1-methyl-2-pyrrolidone (NMP), the free amino groups of the resin were *o-*NBS protected. A mixture of 4-nitrobenzenesulfonyl chloride (*o-*NBS-Cl) and *sym*-collidine in NMP was shaken with the resin beads for 2 hours at room temperature, the process was repeated. The resin was washed with NMP and THF. Subsequently the solution of triphenylphosphine in dry THF and methanol was added to the resin, after 2 minutes the diisopropyl azodicarboxylate (DIAD) in THF was introduced dropwise to the reaction vessel, in order to control exothermic reaction. The reaction was carried out for 2 hours and repeated. The resin was washed with THF and NMP. Finally, the *o-*NBS protecting group was removed by using 2-mercaptoethanol and 1,8-diazabicyclo[5.4.0]undec-7-ene (DBU) in NMP, which were shaken with the resin for 2 hours, and the reaction was repeated.

Modified PS-Sieber resin was loaded manually with Fmoc-ʟ-Ala-OH or Fmoc-ᴅ-Ala-OH (4 equiv), 1-hydroxy-7-azabenzotriazole (HOAt, 4 equiv), *O*-(7-azabenzotriazol-1-yl)-*N,N,N’,N’*-tetramethyluronium hexafluorophosphate (HATU, 4 equiv), *N,N*-diisopropylethylamine (DIEA, 8 equiv) in NMP for 2 hours at room temperature. The loading was analyzed spectrophotometrically by measuring the absorption of the piperidine–dibenzofulvene adduct formed during the Fmoc deprotection (20% v/v piperidine in DMF) at a wavelength of 290 nm. The obtained loadings were quantified to be 0.36–0.60 mmol/g, while the original loading was 0.61 mmol/g.

All steps were carried out manually. Fmoc cleavage was performed with piperidine in DMF (20% v/v; 3 × 10 min). The couplings of Fmoc-ʟ-Ala-OH or Fmoc-ᴅ-Ala-OH were achieved by using 4 equivalents of the corresponding amino acid, *O*-(benzotriazol-1-yl)-*N,N,N’,N’*-tetramethyluronium tetrafluoroborate (TBTU, 4 equiv), DIEA (4 equiv) in DMF for 2 hours at room temperature. The glycosylated building blocks of Fmoc-ʟ-Thr(α-ᴅ-Ac_3_GalNAc)-OH or Fmoc-ᴅ-Thr(α-ᴅ-Ac_3_GalNAc)-OH (1.0–1.2 equiv) were pre-activated with HATU (2.8 equiv) in DMF and added to the reaction vessel, followed by HOAt (2.25 equiv) and DIEA (2.75 equiv) in DMF. Coupling was carried out at room temperature for 3 hours. After final Fmoc deprotection, the amino groups of peptides were acetylated using acetic anhydride (50 equiv), DIEA (12.5 equiv), 1-hydroxybenzotriazole (HOBt,1 equiv) in DMF at room temperature for 40 min. Subsequently, the carbohydrate moieties were deacetylated using 1.0 M hydrazine in dry THF at room temperature for 2 hours. The peptides were cleaved from the resin by treatment with 7–10% TFA in DCM (10 × 5 min). Toluene was added to the mixture in order to avoid high TFA concentrations during evaporation of the cleavage cocktail. The products were precipitated in cold diethyl ether, if possible. The crude products were lyophilised in a mixture of water and acetonitrile (80:20 v/v) and purified by preparative RP-HPLC and identified by Accurate Mass Measurements performed on Agilent Techn. 6220 ToF LCMS; ionisation method: ESI.

**Ac-ʟ-Ala-ʟ-Thr(α-ᴅ-GalNAc)-ʟ-Ala-NH-Me (1)** (27.7 mg, 79.0%); C_21_H_37_N_5_O_10_; exact mass: 519.25 g/mol; ESIMS: [M + H]^+^ 520.2618; ^1^H NMR (600 MHz, DMSO-*d*_6_) δ [ppm] 1.11 (d, *J* = 6.3 Hz, 3H, H^γ^ Thr^2^), 1.19 (d, *J* = 7.0 Hz, 3H, H^β^ Ala^3^), 1.24 (d, *J* = 7.0 Hz, 1H, H^β^ Ala^1^), 1.85 (s, 3H, Ac N-terminal), 1.89 (s, 3H, CH_3_^Ac^ Gal), 2.55 (d, *J* = 4.4 Hz, 3H, Me C-terminal), 3.46 (dd, *J* = 10.8, 5,7 Hz, 1H, CH_2_^C6(1)^ Gal), 3.52 (dd, *J* = 10.7, 6,6 Hz, 1H, CH_2_^C6(2)^ Gal), 3.61 (dd, br, 1H, CH^C3^ Gal), 3.66 (m, 1H, CH^C5^ Gal), 3.72 (m, 1H, CH^C4^ Gal), 4.01 (m, 1H, CH^C2^ Gal), 4.19–4.21 (ov, 2H, H^β^ Thr2, H^α^ Ala^3^), 4.33 (d, *J* = 9.0 Hz, 1H, H^α^ Thr^2^), 4.50 (m, 3H, H^α^ Ala^1^), 4.66 (d, *J* = 3.3 Hz, 1H, CH^C1^ Gal), 7.26 (d, *J* = 9.1 Hz, 1H, NH Gal), 7.81–7.83 (overlay, 2H, NH C-terminal, HN Thr^2^), 8.00 (d, *J* = 7.1 Hz, 1H, HN Ala^3^), 8.18 (d, *J* = 7.4 Hz, 1H, HN Ala^1^); ^13^C NMR (150 MHz, DMSO-*d*_6_) δ [ppm] 18.2 (C^β^ Ala^1^), 18.6 (C^β^ Ala^3^), 19.0 (C^γ^ Thr^2^), 22.8 (C^Ac^ N-terminal), 23.3 (C^Ac^ Gal), 25.9 (Me C-terminal), 48.4 (C^α^ Ala^1^), 48.5 (C^α^ Ala^3^), 49.9 (C^2^ Gal), 56.4 (C^α^ Thr^2^), 61.1 (C^6^ Gal), 68.8 (C^4^ Gal), 69.2 (C^3^ Gal), 72.2 (C^5^ Gal), 74.5 (C^β^ Thr^2^), 98.9 (C^1^ Gal), 169.8 (C(O)^(Ac)^ N-terminal), 170.1 (C(O) Thr^2^), 170.6 (C(O) Gal), 172.8 (C(O) Ala^3^), 173.6 (C(O) Ala^1^).

**Ac-ᴅ-Ala-ᴅ-Thr(α-ᴅ-GalNAc)-ᴅ-Ala-NH-Me (2)** (3.8 mg, 43.3%); C_21_H_37_N_5_O_10_; exact mass: 519.25 g/mol; ESIMS: [M + H]^+^ 520.2647; ^1^H NMR (600 MHz, DMSO-*d*_6_) δ [ppm] 0.94 (d, *J* = 6.2 Hz, 3H, H^γ^ Thr^2^), 1.22 (d, *J* = 7.0 Hz, 3H, H^β^ Ala^3^), 1.27 (d, *J* = 7.2 Hz, 3H, H^β^ Ala^1^), 1.84 (s, 3H, Ac N-terminal), 1.89 (s, 3H, CH_3_^Ac^ Gal ), 2.59 (d, *J* = 4.6 Hz, 3H, Me C-terminal), 3.22 (td, *J* = 9.3, 4.8 Hz, 1H, CH^C3^ Gal), 3.31 (dt, *J* = 9.8, 3.1 Hz, 1H, CH^C5^ Gal), 3.47 (m, 1H, CH^C3^ Gal), 3.52 (m, 2H, CH_2_^C6^ Gal), 3.69 (m, 1H, CH^C2^ Gal), 4.15 (dk, *J* = 6.2, 2.3 Hz, 1H, H^β^ Thr^2^), 4.21–4.27 (m, 1H, H^α^ Ala^3^), 4.31 (dd, *J* = 9.2, 2.2 Hz, 1H, H^α^ Thr^2^), 4.35–4.41 (m, 1H, H^α^ Ala^1^), 4.51 (t, *J* = 5.8 Hz, 1H, HO^C6^ Gal), 4.67 (d, *J* = 3.7 Hz, 1H, CH^C1^ Gal), 4.83 (d, *J* = 5.5 Hz, 1H, HO^C3^ Gal), 4.99 (d, *J* = 5.0 Hz, 1H, HO^C4^ Gal), 7.66 (d, *J* = 7.5 Hz, 1H, HN Ala^3^), 7.69 (d, *J* = 9.2 Hz, 1H, HN Gal), 7.86 (d, *J* = 9.2 Hz, 1H, HN Thr^2^), 7.99 (q, *J* = 4.5 Hz, 1H, HN C-terminal), 8.21 (d, *J* = 7.0 Hz, 1H, HN Ala^1^); ^13^C NMR (150 MHz, DMSO-*d*_6_) δ [ppm] 14.3 (C^γ^ Thr^2^), 18.0 (C^β^ Ala^1^), 19.7 (C^β^ Ala^3^), 23.0 (C^Ac^ Gal), 26.2 (Me C-terminal), 48.7 (C^α^ Ala^3^), 48.9 (C^α^ Ala^1^), 53.8 (C^2^ Gal), 57.4 (Cα Thr^2^), 60.7 (C^6^ Gal), 69.7 (C^β^ Thr^2^), 70.6 (C^4^ Gal), 71.1 (C^3^ Gal), 72.9 (C^5^ Gal), 92.9 (C^1^ Gal), 169.2 (C(O) Thr^2^), 170.3 (C(O)^Ac^ N-terminal), 170.5 (C(O) Gal), 172.9 (C(O) Ala^3^), 173.6 (C(O) Ala^1^).

**Ac-ʟ-Ala-ʟ-Ala-ʟ-Thr(α-ᴅ-GalNAc)-ʟ-Ala-ʟ-Ala-NH-Me (3)** (30.5 mg, 63.3%); C_27_H_47_N_7_O_12_; exact mass: 661.33 g/mol; ESIMS: [M + H]^+^ 662.3376; ^1^H NMR (600 MHz, DMSO-*d*_6_) δ [ppm] 1.09 (d, *J* = 6.4 Hz, 3H, H^γ^ Thr^3^), 1.16 (d, *J* = 7.2 Hz, 3H, H^β^ Ala^2^), 1.18 (d, *J* = 6.7 Hz, 3H, H^β^ Ala^1^), 1.21 (d, *J* = 7.2 Hz, 3H, H^β^ Ala^4^), 1.26 (d, *J* = 7.1 Hz, 3H, H^β^ Ala^5^), 1.84–1.85 (ov, 6H, CH_3_^Ac^ Gal, Ac N-terminal), 2.56 (d, *J* = 4.8 Hz, 3H, Me C-terminal), 3.45 (dd, *J* = 10.9, 6.5 Hz, 1H, CH_2_^C6(1)^ Gal), 3.50 (dd, *J* = 10.9, 5.9 Hz, 1H, CH_2_^C6(2)^ Gal), 3.59 (dd, *J* = 10.9, 2.9 Hz, 1H, CH^C3^ Gal), 3.65 (m, 1H, CH^C5^ Gal), 3.70 (m, 1H, CH^C4^ Gal), 3.98 (m, 1H, CH^C2^ Gal), 4.15 (m, 1H, H^β^ Thr^3^), 4.24 (m, 1H, H^α^ Ala^4^), 4.29 (m, 3H, H^α^ Ala^1^), 4.35–4.37 (ov, 3H, H^α^ Ala^2^, H^α^ Thr^3^, H^α^ Ala^5^), 4.69 (d, *J* = 3.7 Hz, 1H, CH^C1^ Gal), 7.26 (d, *J* = 9.0 Hz, 1H, NH Gal), 7.63 (d, *J* = 7.3 Hz, 1H, HN Thr^3^), 7.76 (q, *J* = 4.5 Hz, 1H, NH C-terminal), 7.92 (d, *J* = 7.4 Hz, 1H, HN Ala^1^), 8.04 (d, *J* = 7.4 Hz, 1H, HN Ala^2^), 8.10 (d, *J* = 7.0 Hz, 1H, HN Ala^4^), 8.17 (d, J = 7.0 Hz, 1H, HN Ala^5^); ^13^C NMR (150 MHz, DMSO-*d*_6_) δ [ppm] 18.4 (C^β^ Ala^5^), 18.7(C^β^ Ala^2^), 18.7 (C^β^ Ala^4^), 18.8 (C^β^ Ala^1^), 18.9 (C^γ^ Thr^3^), 23.1 (C^Ac^ N-terminal), 23.1 (C^Ac^ Gal), 26.0 (Me C-terminal), 48.3 (C^α^ Ala^1^), 48.7 (C^α^ Ala^2^), 48.8 (C^α^ Ala^4^), 48.9 (C^α^ Ala^5^), 50.0 (C^2^ Gal), 56.5 (C^α^ Thr^3^), 61.1 (C^6^ Gal), 68.7 (C^4^ Gal), 69.4 (C^3^ Gal), 72.0 (C^5^ Gal), 75.0 (C^β^ Thr^3^), 99.4 (C^1^ Gal), 170.0 (C(O)^(Ac)^ N-terminal), 170.6 (C(O) Gal), 172.2 (C(O) Ala^1^), 172.6 (C(O) Ala^4^), 172.8 (C(O) Ala^2^), 173.0 (C(O) Ala^5^), 173.2 (C(O) Thr^3^).

**Ac-ᴅ-Ala-ᴅ-Ala-ᴅ-Thr(α-ᴅ-GalNAc)-ᴅ-Ala-ᴅ-Ala-NH-Me (4)** (5.7 mg, 54.3%); C_27_H_47_N_7_O_12_; Exact mass: 661.33 g/mol; ESIMS: [M + H]^+^ 662.3391; ^1^H NMR (600 MHz, DMSO-*d*_6_) δ 0.95 (d, *J* = 6.2 Hz, 3H, H^γ^ Thr^3^), 1.16 (d, *J* = 7.3 Hz, 3H, H^β^ Ala^1^), 1.21 (d, *J* = 7.0 Hz, 3H, H^β^ Ala^5^), 1.22 (d, *J* = 6.7 Hz, 3H, H^β^ Ala^4^), 1.28 (d, *J* = 7.0 Hz, 3H, H^β^ Ala^2^), 1.82 (s, 3H, Ac N-terminal), 1.88 (s, 3H,CH_3_^Ac^ Gal), 2.59 (d, *J* = 4.4 Hz, 3H, Me C-terminal), 3.42 (dd, *J* = 10.4, 6.0 Hz, 1H CH^C6(1)^ Gal), 3.53 (dd, *J* = 10.4, 6.6 Hz, 1H, CH^C6(2)^ Gal), 3.60 (t, *J* = 6.5 Hz, 1H, 1H, CH^C5^ Gal), 3.65 (dd, *J* = 11.2, 2.9 Hz, 1H, 1H, CH^C3^ Gal), 3.70 (d, *J* = 2.3 Hz, 1H, 1H CH^C4^ Gal), 4.04–4.15 (m, 2H, 1H CH^C2^ Gal, H^β^ Thr^3^), 4.19–4.26 (m, 1H, H^α^ Ala^5^), 4.26–4.32 (m, 1H, H^α^ Ala^1^), 4.33–4.42 (m, 3H, H^α^ Thr^3^, H^α^ Ala^4^, H^α^ Ala^2^), 4.67 (d, *J* = 3.5 Hz, 1H, CH^C1^ Gal), 7.52 (d, *J* = 9.1 Hz, 1H, NH Gal), 7.66 (d, *J* = 7.0 Hz, 1H, NH Ala^4^), 7.77–7.84 (m, 2H, NH C-termina + NH Thr^3^), 8.05 (d, *J* = 7.5 Hz, 1H, NH Ala^1^), 8.10 (d, *J* = 7.0 Hz, 1H, NH Ala^2^), 8.24 (d, *J* = 7.6 Hz, 1H, NH Ala^5^); ^13^C NMR (150 MHz, DMSO-*d*_6_) δ [ppm] 14.3 (C^γ^ Thr^3^), 18.2 (C^β^ Ala^2^), 18.7 (C^β^ Ala^1^), 18.7(5) (C^β^ Ala^5^), 19.3 (C^β^ Ala^4^), 22.9 (C^Ac^ N-terminal), 23.4 (C^Ac^ Gal), 26.0 (Me C-terminal), 48.3 (C^α^ Ala^1^), 48.3(5) (C^α^ Ala^4^), 48.7 (C^α^ Ala^2^), 48.7(5) (C^α^ Ala^5^), 49.7 (C^2^ Gal), 57.2 (C^α^ Thr^3^), 60.8 (C^6^ Gal), 67.9 (C^3^ Gal), 68.6 (C^4^ Gal), 69.4 (C^β^ Thr^3^), 71.3 (C^5^ Gal), 93.1 (C^1^ Gal), 169.1 (C(O) Thr^3^), 169.5 (C(O)^Ac^ N-terminal), 170.0 (C(O) Gal), 172.2 (C(O) Ala^4^), 172.7 (C(O) Ala^5^), 172.8 (C(O) Ala^1^), 173.2 (C(O) Ala^2^).

## Supporting Information

File 1 Supplemental materials.
