# Supplementary material for: Synthesis and conformational preferences of short analogues of antifreeze glycopeptides (AFGP)
Source: Beilstein J Org Chem. 2019 Jul 16;15:1581–91. doi: 10.3762/bjoc.15.162 (PMC6664394; doi:10.3762/bjoc.15.162)
Supplement: File 1 — Supplemental materials. [file Beilstein_J_Org_Chem-15-1581-s001.pdf]

BEILSTEIN JOURNAL OF ORGANIC CHEMISTRY

Supporting Information

for

Synthesis and conformational preferences of short analogues of antifreeze glycopeptides (AFGP)

Małgorzata Urbańczyk, Michał Jewgiński, Joanna Krzciuk-Gula, Jerzy Góra, Rafał Latajka and Norbert Sewald

Beilstein J. Org. Chem. **2019,** *15,* 1581–1591. doi:10.3762/bjoc.15.162

Supplemental materials

License and Terms: This is a supporting information file under the terms of the Creative Commons Attribution License (http://creativecommons.org/licenses/by/4.0). Please note that the reuse, redistribution and reproduction in particular requires that the authors and source are credited.
The license is subject to the *Beilstein Journal of Organic Chemistry* terms and conditions: (https://www.beilstein-journals.org/bjoc)

General methods:

All air- and moisture-sensitive reactions were carried out in a dry argon atmosphere in flame-dried glass flasks. Dichloromethane was freshly distilled from CaH_2 and toluene from Na. DMF was distilled from ninhydrine. All amino acids and the Fmoc-Sieber PS resin were purchased from Iris Biotech (Marktredwitz, Germany). Other chemicals were bought from Sigma Aldrich (Hamburg, Germany), Acros (Geel, Belgium), and VWR (Darmstadt, Germany). All chemicals were used as purchased, if not stated otherwise.

All reactions were monitored by TLC performed on silica gel 60 precoated aluminium foil (Merc). Reagents and solvents were purchased at Acros, Sigma Aldrich, Fluorochem and Iris Biotech in a p.a. quality and used without further treatment.

Analytical Reverse Phase High Performance Liquid Chromatography (RP-HPLC) was carried out on a Thermo Separation Products system consisting of a UV 6000 diode array detector and a P 4000 pump equipped with a Phenomenex HPLC guard cartridge system (C12; 4 x 3.00 mm) and a Phenomenex Jupiter 4 µ Proteo 90 Å column (C12; 250 x 4.60 mm). Flow rate 1 mL min^{-1}. Eluent A: $H_2O/CH_3CN/TFA$ (95/4.9/0.1), Eluent B: $CH_3CN/H_2O/TFA$ (95/4.9/0.1). Preparative RP-HPLC was carried out with a Thermo Separation Products system consisting of a UV-1000 detector and a P-4000 pump equipped with a Vydac high-performance guard column (C18) and a Phenomenex Jupiter 10 µ Proteo 90 Å column (C12; 250 x 21.20 mm) or with a Hitachi MERCK LaChrom system consisting of a UV-Vis L-7420 detector and a L7150 pump equipped with a Vydac high-performance guard column (C18) and a Phenomenex Jupiter 10 µ 300 Å column (C18; 250 x 21.20 mm). Flow rate 7.5 mL min^{-1}. Eluent A: $H_2O/CH_3CN/TFA$ (95/4.9/0.1), Eluent B: $CH_3CN/H_2O/TFA$ (95/4.9/0.1).

Table S1. Preparative HPLC method.

Time [min]	A [%]	B [%]	Flow [ml/min]
0	0	100	10
5	0	100	10
35	100	0	10
40	100	0	10
45	0	100	10

Table S2. Analytical PR-HPLC method.

Time [min]	A [%]	B [%]	Flow [µl/min]
0.0	0	100	700
5.0	100	0	700
6.0	0.0	100	700
6.5	0.0	100	700

Matrix Assisted Laser Desorption/Ionization – Time of Flight

Mass spectra were recorded on Voyager DE Instrument (PE Biosystems), using 2,5-dihydroxybenzoic acid as a matrix.

Accurate Mass Measurements

Accurate mass meassurements were performed on Agilent Techn. 6220 TOF LCMS; Ionisation Method: ESI.

Nuclear Magnetic Resonance Spectroscopy

NMR spectra were measured on Bruker DRX 500 at 500MHz or 600MHz (^{1}H) and 600MHz (^{13}C).

Reactions:

Preparation of 3,4,6-tri-O-acetyl-D-galactal

3,4,6-tri-*O*-acetyl-D-galactal was obtained from D-(+)-galactose (1eq.), acetic anhydride (5.07eq.), Et_3N (5.07eq.) and DMAP (0.01eq.) in methylene chloride as a solvent in an overnight reaction at room temperature. The crude product was washed with water, 1M HCl, sat. $NaHCO_3$ and NaCl solution. The organic layer was dried over Na_2SO_4 and DCM was removed in vacuo resulting *β*-D-galactose pentaacetate. The product was dissolved in DCM and HBr 33% in AcOH was added dropwise to the solution at 0°C, maintaining this temperature conditions for an hour longer and left overnight at room temperature until the starting material was completely consumed according to TLC. The reaction solution was diluted with DCM, washed with ice-cold water, 1% aq. $NaHCO_3$, sat. $NaHCO_3$ 3 times and NaCl solutions. Organic layer was dried over Na_2SO_4 and solvent was evaporated to give 2,3,4,6-tetra-*O*-acetyl-*α*-bromo-D-galactose. The crude product (1eq) was dissolved in glacial acetic acid, after cooling the mixture to 0°C, $CuSO_4$ (0.43eq) in water and activated (*I*) zinc dust (15.5eq) were added. The reaction conditions were maintained for 30 min, afterwards the suspension was stirred overnight at room temperature.

The mixture was filtered through Celite and the filter cake was washed 5 times with water and 3 times with DCM. The aqueous layer was additionally extracted with DCM. Then combined organic layers were washed with sat. $NaHCO_3$, water and dried over Na_2SO_4. After evaporation of the solvents the 3,4,6-tri-*O*-acetyl-D-galactal was synthesized.

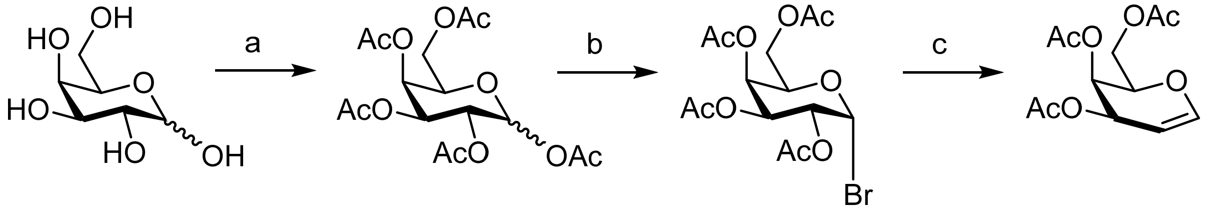

Scheme S1. Preparation of 3,4,6-tri-*O*-acetyl-D-galactal, where: a) Ac_2O, Et_3N, DMAP, CH_2Cl_2, rt, overnight; b) HBr, CH_2Cl_2, 0°C – rt, overnight; c) Zn dust, $CuSO_4 \cdot 5H_2O$, AcOH, 0°C – rt, overnight

Azidochlorination of Glycals

To a solution of the 3,4,6-tri-*O*-acetyl-D-galactal (1.0eq.) in acetonitrile, p. a. (80 mL, 8mL mmol−1) at -30 °C, ferric chloride hexahydrate (0.8eq.), sodium azide (1.1eq.) and hydrogen peroxide (1.1eq.; 33 % aq. solution) were added. The reaction was stirred in this temperature overnight. Then the mixture was diluted with diethyl ether and washed with water, sat. $NaHCO_3$ and NaCl until the organic layer was decolorized. The organic layer was dried over $MgSO_4$ and the solvent was evaporated. To avoid the loss of 3,4,6-tri-*O*-acetyl-2-azido-2-desoxy-α-D-galactopyranosyl chloride the chromatographic purification has been omitted.

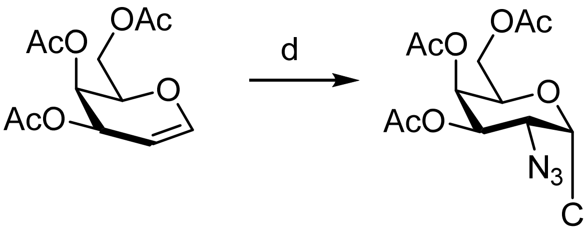

Scheme S2. Preparation of Azidochlorination of Glycals, where d) $FeCl_3 \cdot H_2O$, NaN_3, H_2O_2, CH_3CN, -30°C, overnight.

Glycosylation using Glycosyl Halides

The mixture of Fmoc-Thr-OAllyl (1.0 eq), Ag_2CO_3 (1.5 eq) and freshly activated 4Å molecular sieve in toluene and DCM (1:2) was stirred at -10 °C for 30 min. Then $AgClO_4$ (0.25 eq), dissolved in toluene, was slowly added. Afterwards 3,4,6-Tri-*O*-acetyl-2-azido-2-desoxy-α-D-galactopyranosyl chloride (1.5 eq) in a mixture of toluene and DCM (1:1) was gradually added to the solution. The reaction mixture was stirred in the dark under argon at room temperature overnight. After diluting with DCM the suspension was filtered through Celite, washed with water and sat. $NaHCO_3$ solution. The organic layer was dried over Na_2SO_4, the solvent was removed in the vacuo and the crude product purified by column chromatography.

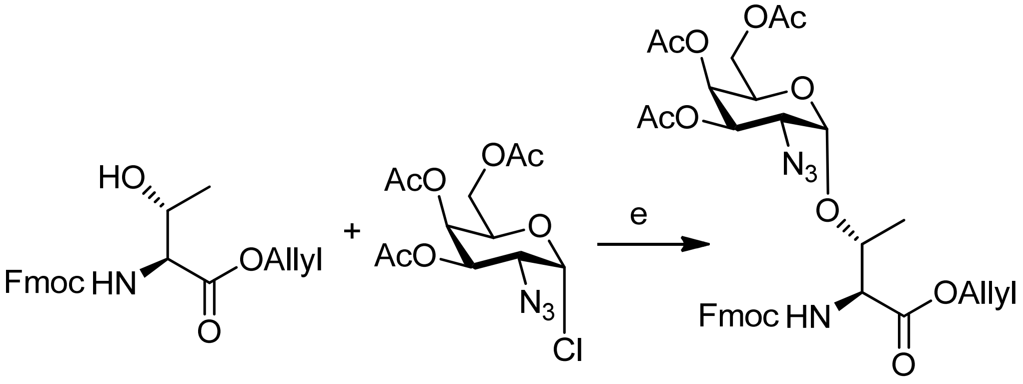

Scheme S3. Glycolysation of threonine residue where e) $AgClO_4$, Ag_2CO_3, CH_2Cl_2, toluene, -10°C (30min) – rt, in the dark with argon, overnight

Reduction of the azido group on carbon C2 of the sugar was carried out with thioacetic acid and pyridine (2:1) stirred at 50°C for 30min. The reaction mixture was directly purified on a chromatographic column (PE:EtOAc, 1:1 → 1:2)

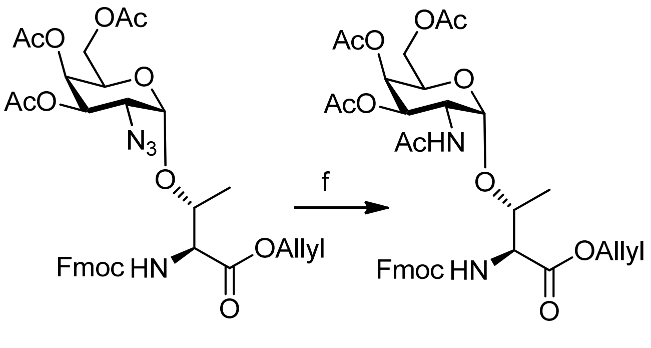

Scheme S4. Reduction of the azido group on carbon C2, where f) pyridine, AcSH , 50°C; 30min.

Ready building block, used in SPPS, was obtained by removal of the allyl group with the palladium catalyst (0.03eq.) and phenylsilane (1.1eq.) as a scavenger in CH_2Cl_2. Subsequently the mixture was washed with NH_4Cl solution and water. Organic layer was dried over Na_2SO_4 and solvent was removed in vacuum. Crude product was purified on column (DCM:MeOH:AcOH; 95:4.5:0.5).

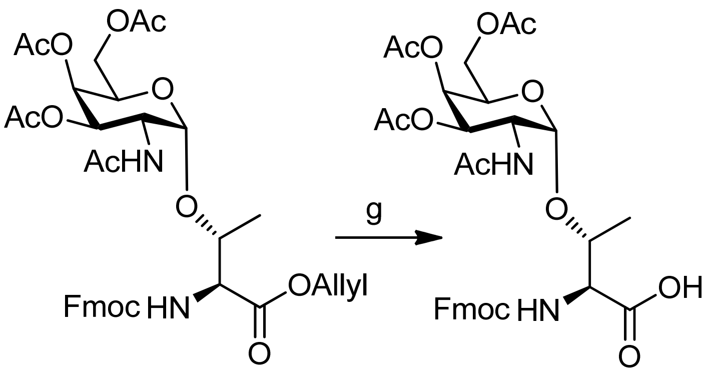

Scheme S5. Reaction of removing of allyl group from the ready glycosylated building block, where g) $Pd(PPh_3)_4$, PhSiH, CH_2Cl_2, rt, in the dark, 2 – 4h.

Building Blocks:

Fmoc-L-Thr(α-D-Ac₃GalNAc)-OH

^{1}H NMR (600 MHz, DMSO-d_6): δ [ppm] = 1. 13 (d, J = 6.3 Hz, 3H, $CH_3{}^{γ}$ Thr); 1,81 (s, 3H, $CH_3{}^{Ac}$ Gal); 1,92 (s, 3H, $CH_3{}^{Ac}$ Gal); 2.00 (s, 3H, $CH_3{}^{Ac}$ Gal); 2.10 (s, 3H, $CH_3{}^{Ac}$ Gal); 3.83 (m, 1H, $CH_2{}^{C6}$ Gal); 4.03 (m, 1H, $CH_2{}^{C6}$ Gal); 4.23-4.31 (ov, 7H, CH Fmoc, CH_2 Fmoc, $H^{α}$ Thr, $CH^{β}$ Thr, CH^{C3} Gal, CH^{C5} Gal); 4.60 (d, J = 8.4 Hz, 1H, CH^{C4} Gal); 5.02 (dd, J = 11.4, 3.4, 1H, CH^{C2} Gal); 5.23 (d, J = 3.3 Hz, 1H, CH^{C1} Gal); 7.34 (m, 2H, $C^{arom}{}_{Fmoc}$); 7.43 (m, 2H, $C^{arom}{}_{Fmoc}$); 7.76 (m, 2H, $C^{arom}{}_{Fmoc}$); 7.90 (ov, 2H, $C^{arom}{}_{Fmoc}$); 7.93 (ov, 1H, NH_{Thr}).
$C_{33}H_{38}N_2O_{13}$
M = 670.67 g/mol
Exact mass: 670.24
MS (MALDI-ToF) = 671,2 $[M + H]^+$, 693,6 $[M + Na]^+$, 709,2 $[M + K]^+$

Fmoc-D-Thr(α-D-Ac₃GalNAc)-OH

^{1}H NMR (600 MHz, DMSO-d_6): δ [ppm] = 1.01 (d, J = 6.0 Hz, 3H, $CH_3{}^{γ}$ Thr); 1.86 (s, 3H, $CH_3{}^{Ac}$ Gal); 1.92 (s, 3H, $CH_3{}^{Ac}$ Gal); 1.95 (s, 3H, $CH_3{}^{Ac}$ Gal); 2.09 (s, 3H, $CH_3{}^{Ac}$ Gal); 3.92 (dd, J = 10.4, 5.3 Hz, 2H, $CH_2{}^{C6}$ Gal);); 4.09 (m, 1H, $CH_2{}^{C6}$ Gal); 4.23 - 4.35 (ov, 7H, CH Fmoc, CH_2 Fmoc, $H^{α}$ Thr, $CH^{β}$ Thr, CH^{C3} Gal, CH^{C5} Gal); 4.87 (m, 1H, CH^{C4} Gal); 4.96 (m, 1H, CH^{C2} Gal); 5.27 (s, br. 1H, CH^{C1} Gal); 7.29 (t, J = 7.4 Hz, 1H, $C^{arom}{}_{Fmoc}$); 7.33 (t, J = 7.3 Hz, 1H, $C^{arom}{}_{Fmoc}$); 7.43 (t, J = 7.4 Hz, 2H, $C^{arom}{}_{Fmoc}$); 7.65 (br, NH Gal); 7.74 (d, J = 7.4 Hz, 1H, $C^{arom}{}_{Fmoc}$); 7.79 (d, J = 7.5 Hz, 1H, $C^{arom}{}_{Fmoc}$); 7.90 (d, J = 7.5 Hz, 2H, $C^{arom}{}_{Fmoc}$); 8.07 (d, J = 8.8 Hz, 1H, NH Thr).
$C_{33}H_{38}N_2O_{13}$
M = 670.67 g/mol
Exact mass: 670.24
MS (MALDI-ToF) = 671.1 $[M + H]^+$, 693.3 $[M + Na]^+$, 709.3 $[M + K]^+$

Peptides:

Ac-L-Ala-L-Thr(α-D-GalNAc)-L-Ala-NH-Me (1)

^{1}H NMR (600 MHz, DMSO-d$_6$): δ [ppm] = 1.11 (d, J = 6.3 Hz, 3H, H$^{\gamma}$ Thr2); 1.19 (d, J = 7.0 Hz, 3H, H$^{\beta}$ Ala3); 1.24 (d, J = 7.0 Hz, 1H, H$^{\beta}$ Ala1); 1.85 (s, 3H, Ac N-terminal); 1.89 (s, 3H, CH$_3^{Ac}$ Gal); 2.55 (d, J = 4.4 Hz, 3H, Me C-terminal); 3.46 (dd, J = 10.8, 5,7 Hz, 1H, CH$_2^{C6(1)}$ Gal); 3.52 (dd, J = 10.7, 6,6 Hz, 1H, CH$_2^{C6(2)}$ Gal); 3.61 (dd, br, 1H, CH^{C3} Gal); 3.66 (m, 1H, CH^{C5} Gal); 3.72 (m, 1H, CH^{C4} Gal); 4.01 (m, 1H, CH^{C2} Gal); 4.19-4.21 (ov, 2H, H$^{\beta}$ Thr2, H$^{\alpha}$ Ala3); ,); 4.33 (d, J = 9.0 Hz, 1H, H$^{\alpha}$ Thr2); 4.50 (m, 3H, H$^{\alpha}$ Ala1); 4.66 (d, J = 3.3 Hz, 1H, CH^{C1} Gal); 7.26 (d, J = 9.1 Hz, 1H, NH Gal); 7.81-7.83 (ov, 2H, NH C-terminal, HN Thr2); 8.00 (d, J = 7.1 Hz, 1H, HN Ala3); 8.18 (d, J = 7.4 Hz, 1H, HN Ala1);
^{13}C NMR (600 MHz, DMSO-d$_6$): δ [ppm] = 18.2 (C$^{\beta}$ Ala1); 18.6 (C$^{\beta}$ Ala3); 19.0 (C$^{\gamma}$ Thr2); 22.8 (C^{Ac} N-terminal); 23.3 (C^{Ac} Gal); 25.9 (Me C-terminal); 48.4 (C$^{\alpha}$ Ala1); 48.5 (C$^{\alpha}$ Ala3); 49.9 (C^{2} Gal); 56.4 (C$^{\alpha}$ Thr2); 61.1 (C^{6} Gal); 68.8 (C^{4} Gal); 69.2 (C^{3} Gal); 72.2 (C^{5} Gal); 74.5 (C$^{\beta}$ Thr2); 98.9 (C^{1} Gal); 169.8 (C(O)$^{(Ac)}$ N-terminal); 170.1 (C(O) Thr2); 170.6 (C(O) Gal); 172.8 (C(O) Ala3); 173.6 (C(O) Ala1).
$C_{21}H_{37}N_5O_{10}$
M = 519.55 g/mol
Exact mass: 519.25
MS (ESI) = 520.2618 [M + H]$^+$

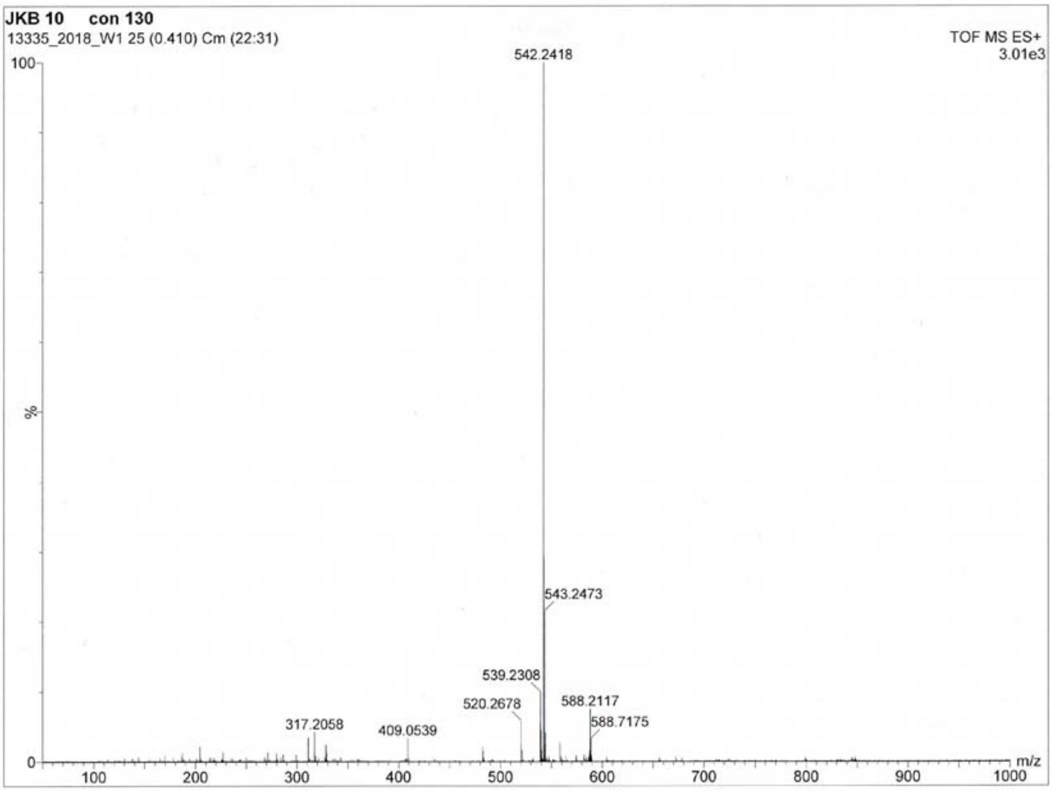

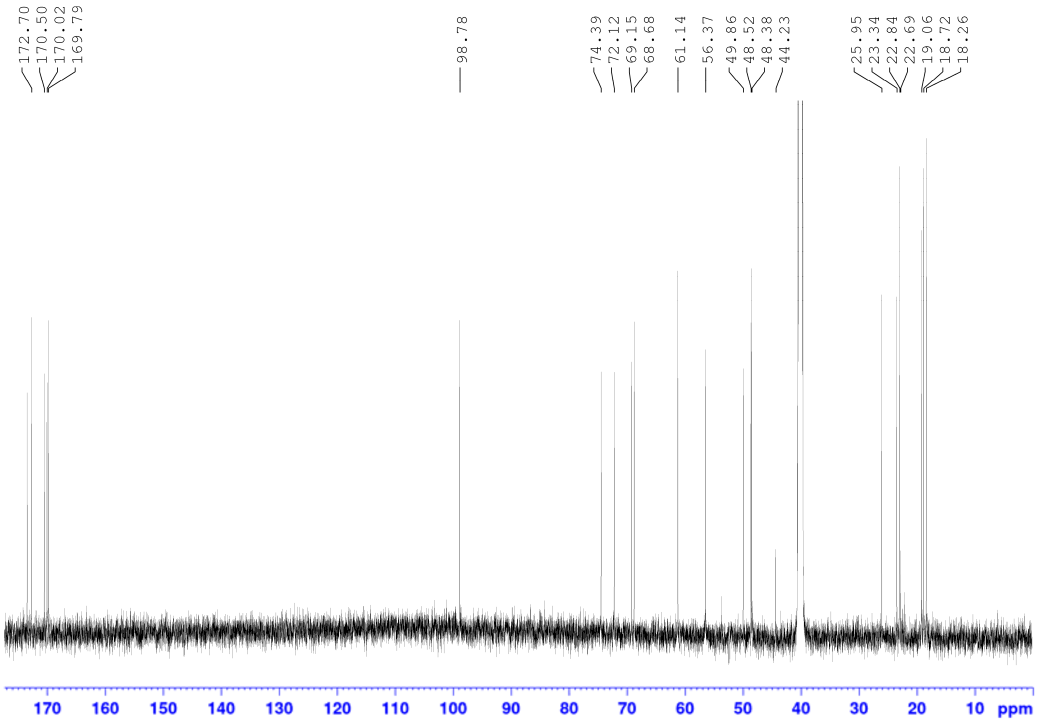
172.70
170.50
170.02
169.79
98.78
74.39
72.12
69.15
68.68
61.14
56.37
49.86
48.52
48.38
44.23
25.95
23.34
22.84
22.69
19.06
18.72
18.26
170 160 150 140 130 120 110 100 90 80 70 60 50 40 30 20 10 ppm

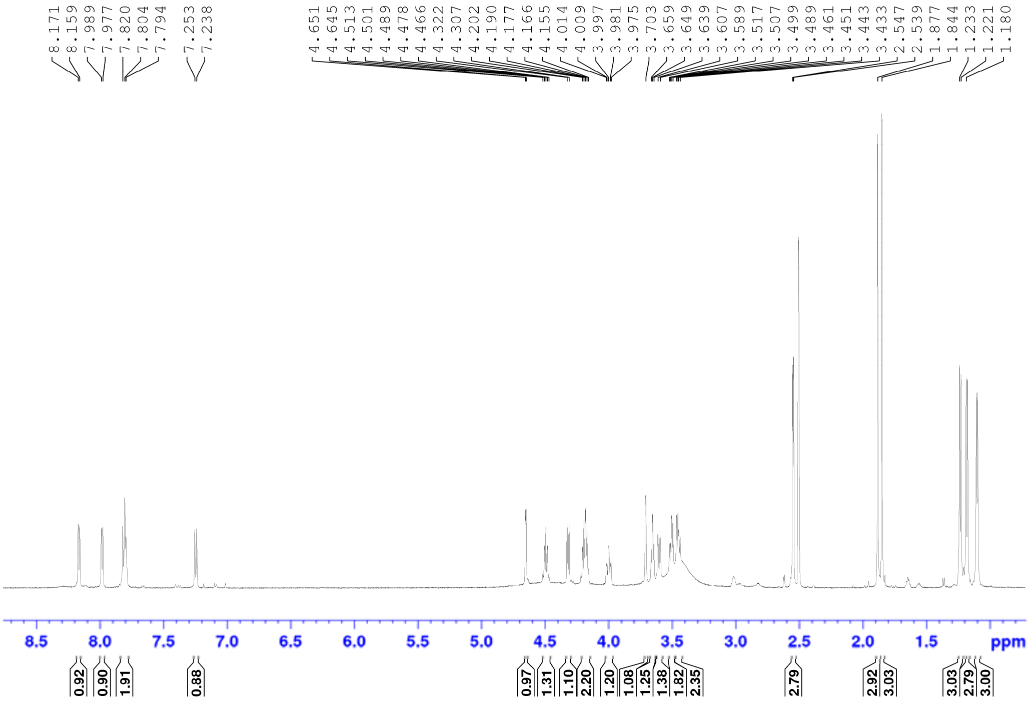
8.171
8.159
7.989
7.977
7.820
7.804
7.794
7.253
7.238
4.651
4.645
4.513
4.501
4.489
4.478
4.466
4.322
4.307
4.202
4.190
4.177
4.166
4.155
4.014
4.009
3.997
3.981
3.975
3.703
3.659
3.649
3.639
3.607
3.589
3.517
3.507
3.499
3.489
3.461
3.451
3.443
3.433
2.547
2.539
1.877
1.844
1.233
1.221
1.180
8.5 8.0 7.5 7.0 6.5 6.0 5.5 5.0 4.5 4.0 3.5 3.0 2.5 2.0 1.5 ppm
0.92
0.90
1.91
0.88
0.97
1.31
1.10
2.20
1.20
1.08
1.25
1.38
1.82
2.35
2.79
2.92
3.03
3.03
2.79
3.00

Ac-D-Ala-D-Thr(α-D-GalNAc)-D-Ala-NH-Me (2)

^{1}H NMR (600 MHz, DMSO-d_6) δ [ppm] = 0.94 (d, J = 6.2 Hz, 3H, H^{γ} Thr2), 1.22 (d, J = 7.0 Hz, 3H, H^{β} Ala3), 1.27 (d, J = 7.2 Hz, 3H, H^{β} Ala1), 1.84 (s, 3H, Ac N-terminal), 1.89 (s, 3H, CH_3^{Ac} Gal), 2.59 (d, J = 4.6 Hz, 3H, Me C-terminal), 3.22 (td, J = 9.3, 4.8 Hz, 1H, CH^{C3} Gal), 3.31 (dt, J = 9.8, 3.1 Hz, 1H, CH^{C5} Gal), 3.47 (m, 1H, CH^{C3} Gal), 3.52 (m, 2H, CH_2^{C6} Gal), 3.69 (m, 1H, CH^{C2} Gal), 4.15 (dk, J = 6.2, 2.3 Hz, 1H, H^{β} Thr2), 4.21-4.27 (m, 1H, H^{α} Ala3), 4.31 (dd, J = 9.2, 2.2 Hz, 1H, H^{α} Thr2), 4.35-4.41 (m, 1H, H^{α} Ala1), 4.51 (t, J = 5.8 Hz, 1H, HO^{C6} Gal), 4.67 (d, J = 3.7 Hz, 1H, CH^{C1} Gal), 4.83 (d, J = 5.5 Hz, 1H, HO^{C3} Gal), 4.99 (d, J = 5.0 Hz, 1H, HO^{C4} Gal), 7.66 (d, J = 7.5 Hz, 1H, HN Ala3), 7.69 (d, J = 9.2 Hz, 1H, HN Gal), 7.86 (d, J = 9.2 Hz, 1H, HN Thr2), 7.99 (q, J = 4.5 Hz, 1H, HN C-terminal), 8.21 (d, J = 7.0 Hz, 1H, HN Ala1)

^{13}C NMR (150MHz, DMSO-d_6) δ [ppm] = 14.3 (C^{γ} Thr2); 18.0 (C^{β} Ala1); 19.7 (C^{β} Ala3); 23.0 (C^{Ac} Gal); 26.2 (Me C-terminal); 48.7 (C^{α} Ala3); 48.9 (C^{α} Ala1); 53.8 (C^{2} Gal); 57.4 (Cα Thr2); 60.7 (C^{6} Gal); 69.7 (C^{β} Thr2); 70.6 (C^{4} Gal); 71.1 (C^{3} Gal); 72.9 (C^{5} Gal); 92.9 (C^{1} Gal); 169.2 (C(O) Thr2); 170.3 ($C(O)^{Ac}$ N-terminal); 170.5 (C(O) Gal); 172.9 (C(O) Ala3); 173.6 (C(O) Ala1)

$C_{21}H_{37}N_5O_{10}$

M = 519.55 g/mol

Exact mass: 519.25

MS (ESI) = 520.2647 $[M + H]^+$

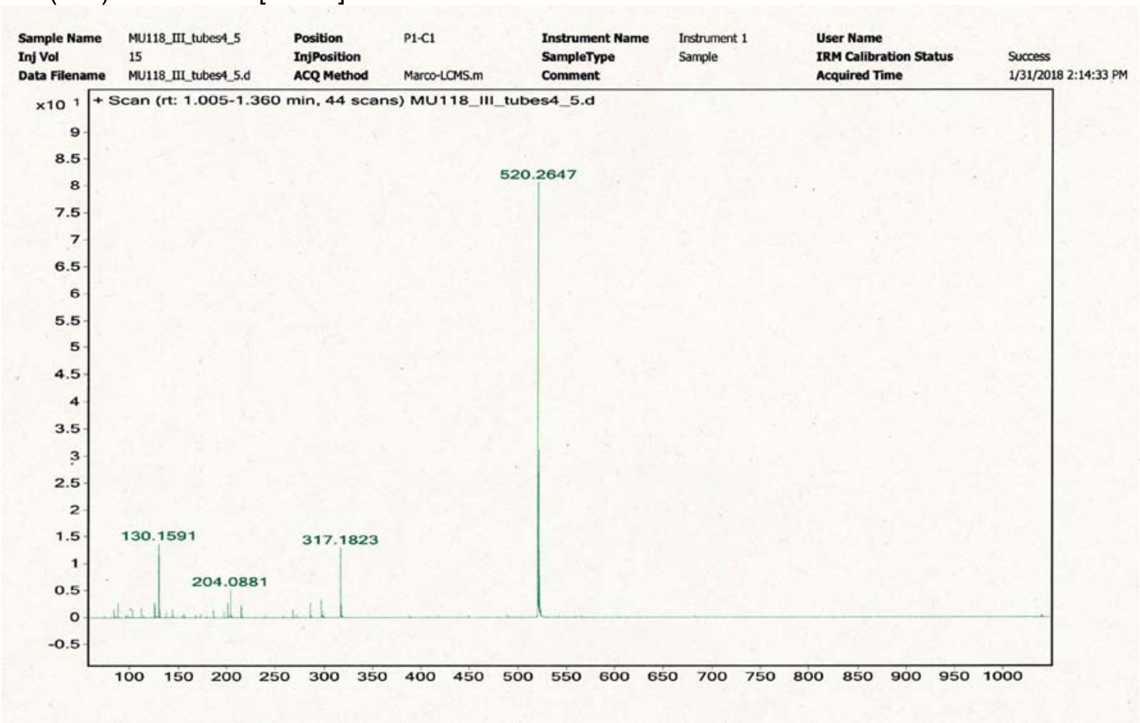

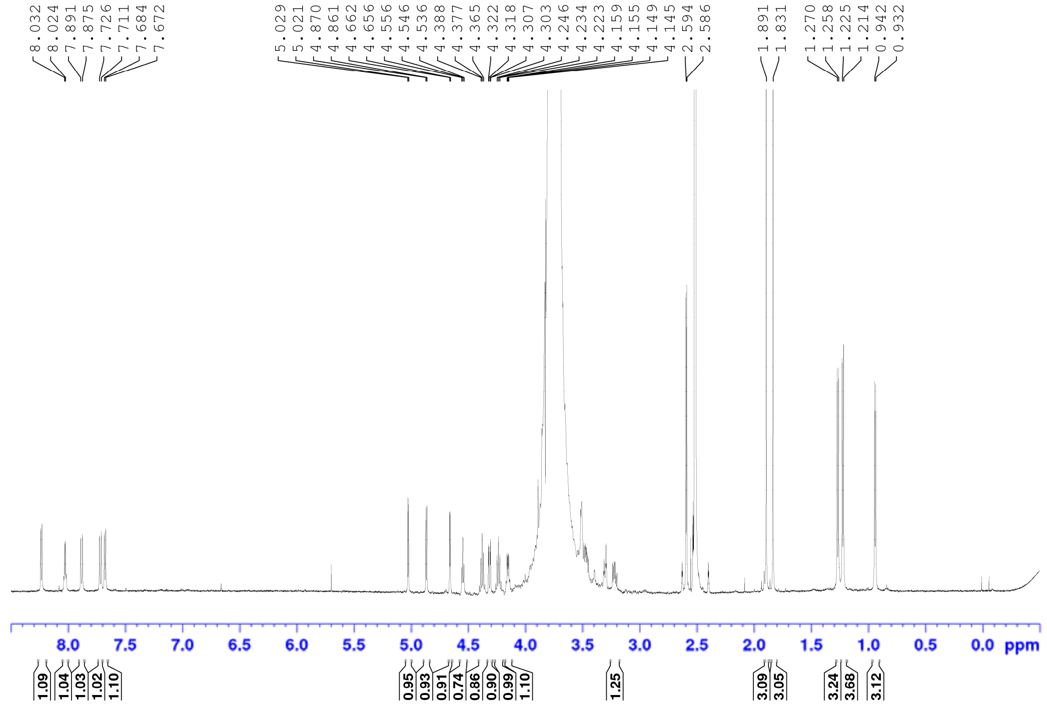
8.032
8.024
7.891
7.875
7.726
7.711
7.684
7.672
5.029
5.021
4.870
4.861
4.662
4.656
4.556
4.546
4.536
4.388
4.377
4.365
4.322
4.318
4.307
4.303
4.246
4.234
4.223
4.159
4.155
4.149
4.145
2.594
2.586
1.891
1.831
1.270
1.258
1.225
1.214
0.942
0.932
8.0 7.5 7.0 6.5 6.0 5.5 5.0 4.5 4.0 3.5 3.0 2.5 2.0 1.5 1.0 0.5 0.0 ppm
1.09
1.04
1.03
1.02
1.10
0.95
0.93
0.91
0.74
0.86
0.90
0.99
1.10
1.25
3.09
3.05
3.24
3.68
3.12

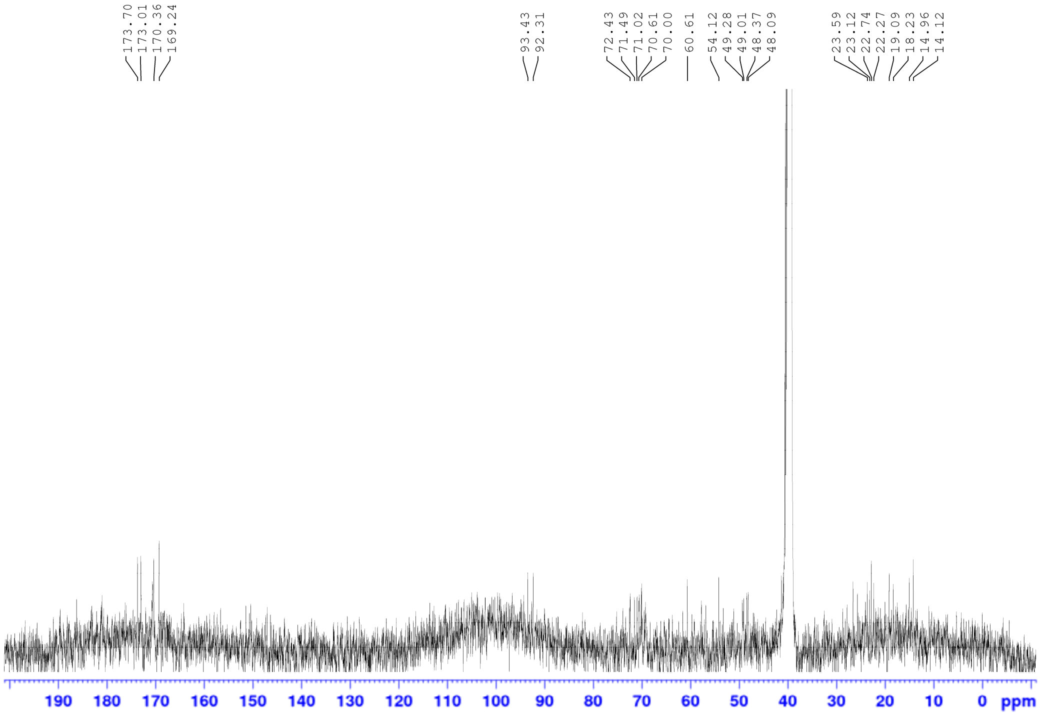
173.70
173.01
170.36
169.24
93.43
92.31
72.43
71.49
71.02
70.61
70.00
60.61
54.12
49.28
49.01
48.37
48.09
23.59
23.12
22.74
22.27
19.09
18.23
14.96
14.12
190 180 170 160 150 140 130 120 110 100 90 80 70 60 50 40 30 20 10 0 ppm

Ac-L-Ala L-Ala-L-Thr(α-D-GalNAc)-L-Ala-L-Ala-NH-Me (3)

^{1}H NMR (600 MHz, DMSO-d_6): δ [ppm] = 1.09 (d, J = 6.4 Hz, 3H, H^{γ} Thr3); 1.16 (d, J = 7.2 Hz, 3H, H^{β} Ala2); 1.18 (d, J = 6.7 Hz, 3H, H^{β} Ala1); 1.21 (d, J = 7.2 Hz, 3H, H^{β} Ala4); 1.26 (d, J = 7.1 Hz, 3H, H^{β} Ala5); 1.84-1.85 (ov, 6H, CH_3^{Ac} Gal, Ac N-terminal); 2.56 (d, J = 4.8 Hz, 3H, Me C-terminal); 3.45 (dd, J = 10.9, 6.5 Hz, 1H, $CH_2^{C6(1)}$ Gal); 3.50 (dd, J = 10.9, 5.9 Hz, 1H, $CH_2^{C6(2)}$ Gal); 3.59 (dd, J = 10.9, 2.9 Hz, 1H, CH^{C3} Gal); 3.65 (m, 1H, CH^{C5} Gal); 3.70 (m, 1H, CH^{C4} Gal); 3.98 (m, 1H, CH^{C2} Gal); 4.15 (m, 1H, H^{β} Thr3); 4.24 (m, 1H, H^{α} Ala4); 4.29 (m, 3H, H^{α} Ala1); 4.35-4.37 (ov, 3H, H^{α} Ala2, H^{α} Thr3, H^{α} Ala5); 4.69 (d, J = 3.7 Hz, 1H, CH^{C1} Gal); 7.26 (d, J = 9.0 Hz, 1H, NH Gal); 7.63 (d, J = 7.3 Hz, 1H, HN Thr3); 7.76 (q, J = 4.5 Hz, 1H, NH C-terminal); 7.92 (d, J = 7.4 Hz, 1H, HN Ala1); 8.04 (d, J = 7.4 Hz, 1H, HN Ala2); 8.10 (d, J = 7.0 Hz, 1H, HN Ala4); 8.17 (d, J = 7.0 Hz, 1H, HN Ala5)

^{13}C NMR (600 MHz, DMSO-d_6): δ [ppm] = 18.4 (C^{β} Ala5); 18.7(C^{β} Ala2); 18.7 (C^{β} Ala4); 18.8 (C^{β} Ala1); 18.9 (C^{γ} Thr3); 23.1 (C^{Ac} N-terminal); 23.1 (C^{Ac} Gal); 26.0 (Me C-terminal); 48.3 (C^{α} Ala1); 48.7 (C^{α} Ala2); 48.8 (C^{α} Ala4); 48.9 (C^{α} Ala5); 50.0 (C^{2} Gal); 56.5 (C^{α} Thr3); 61.1 (C^{6} Gal); 68.7 (C^{4} Gal); 69.4 (C^{3} Gal); 72.0 (C^{5} Gal); 75.0 (C^{β} Thr3); 99.4 (C^{1} Gal); 170.0 ($C(O)^{(Ac)}$ N-terminal); 170.6 (C(O) Gal); 172.2 (C(O) Ala1); 172.6 (C(O) Ala4); 172.8 (C(O) Ala2); 173.0 (C(O) Ala5); 173.2 (C(O) Thr3)

$C_{27}H_{47}N_7O_{12}$

M = 661.71 g/mol

Exact mass: 661.33

MS (ESI) = 662.3376 $[M + H]^{+}$

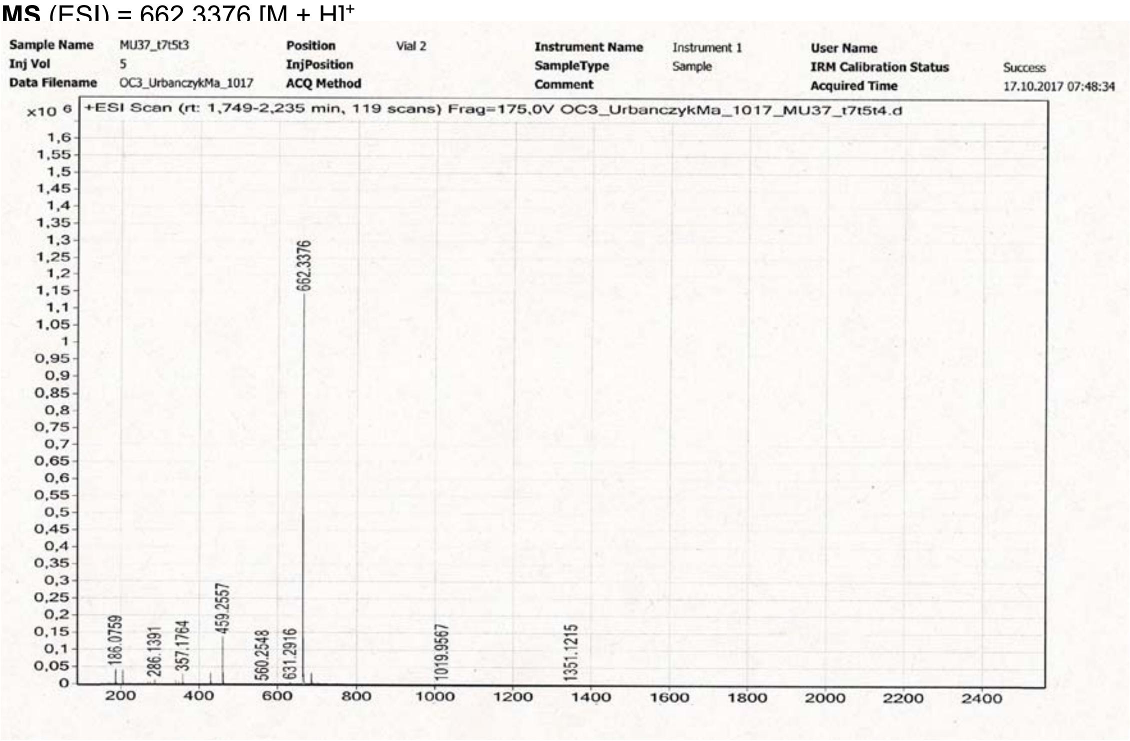

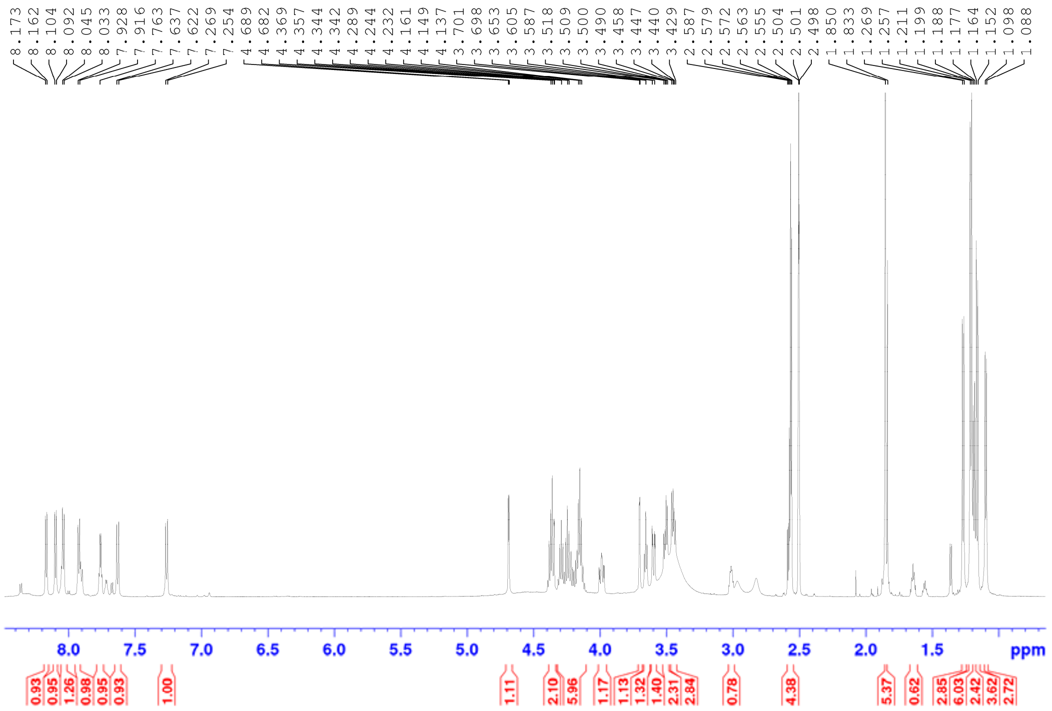
8.173 8.162 8.104 8.092 8.045 8.033 7.928 7.916 7.763 7.637 7.622 7.269 7.254 4.689 4.682 4.369 4.357 4.344 4.342 4.289 4.244 4.232 4.161 4.149 4.137 3.701 3.698 3.653 3.605 3.587 3.518 3.509 3.500 3.490 3.458 3.447 3.440 3.429 2.587 2.579 2.572 2.563 2.555 2.504 2.501 2.498 1.850 1.833 1.269 1.257 1.211 1.199 1.188 1.177 1.164 1.152 1.098 1.088
8.0 7.5 7.0 6.5 6.0 5.5 5.0 4.5 4.0 3.5 3.0 2.5 2.0 1.5 ppm
0.93 0.95 1.26 0.98 0.95 0.93 1.00 1.11 2.10 5.96 1.17 1.13 1.32 1.40 2.31 2.84 0.78 4.38 5.37 0.62 2.85 6.03 2.42 3.62 2.72

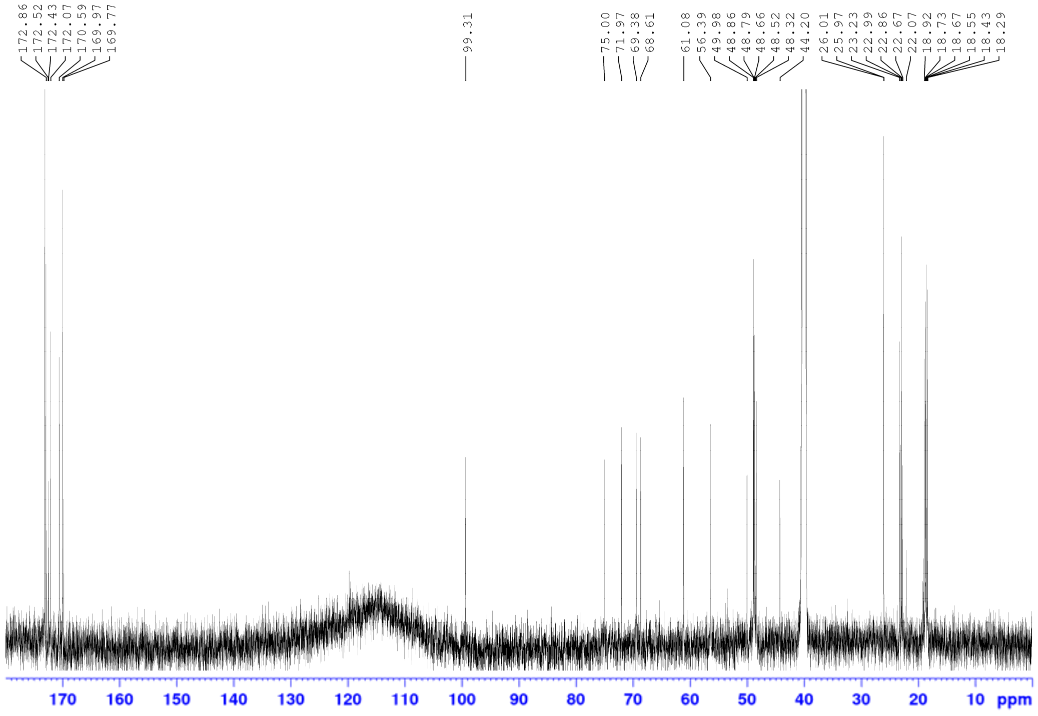
172.86 172.52 172.43 172.07 170.59 169.97 169.77 99.31 75.00 71.97 69.38 68.61 61.08 56.39 49.98 48.86 48.79 48.66 48.52 48.32 44.20 26.01 25.97 23.23 22.99 22.86 22.67 22.07 18.92 18.73 18.67 18.55 18.43 18.29
170 160 150 140 130 120 110 100 90 80 70 60 50 40 30 20 10 ppm

Ac-D-Ala-D-Ala-D-Thr(α-D-GalNAc)-D-Ala-D-Ala-NH-Me (4)

^{1}H NMR (600 MHz, DMSO-D6) δ 0.95 (d, J = 6.2 Hz, 3H, $H^{γ}$ Thr3), 1.16 (d, J = 7.3 Hz, 3H, $H^{β}$ Ala1), 1.21 (d, J = 7.0 Hz, 3H, $H^{β}$ Ala5), 1.22 (d, J = 6.7 Hz, 3H, $H^{β}$ Ala4), 1.28 (d, J = 7.0 Hz, 3H, $H^{β}$ Ala2), 1.82 (s, 3H, Ac N-terminal), 1.88 (s, 3H,CH_3^{Ac} Gal), 2.59 (d, J = 4.4 Hz, 3H, Me C-terminal), 3.42 (dd, J = 10.4, 6.0 Hz, 1H $CH^{C6(1)}$ Gal), 3.53 (dd, J = 10.4, 6.6 Hz, 1H, $CH^{C6(2)}$ Gal), 3.60 (t, J = 6.5 Hz, 1H, 1H, CH^{C5} Gal), 3.65 (dd, J = 11.2, 2.9 Hz, 1H, 1H, CH^{C3} Gal), 3.70 (d, J = 2.3 Hz, 1H, 1H CH^{C4} Gal), 4.04-4.15 (m, 2H, 1H CH^{C2} Gal, $H^{β}$ Thr3), 4.19-4.26 (m, 1H, $H^{α}$ Ala5), 4.26-4.32 (m, 1H, $H^{α}$ Ala1), 4.33-4.42 (m, 3H, $H^{α}$ Thr3 $H^{α}$ Ala4 $H^{α}$ Ala2), 4.67 (d, J = 3.5 Hz, 1H, CH^{C1} Gal), 7.52 (d, J = 9.1 Hz, 1H, NH Gal), 7.66 (d, J = 7.0 Hz, 1H, NH Ala4), 7.77-7.84 (m, 2H, NH C-termina + NH Thr3), 8.05 (d, J = 7.5 Hz, 1H, NH Ala1), 8.10 (d, J = 7.0 Hz, 1H, NH Ala2), 8.24 (d, J = 7.6 Hz, 1H, NH Ala5)
^{13}C NMR (150MHz, DMSO-d_6) δ [ppm] = 14.3 ($C^{γ}$ Thr3); 18.2 ($C^{β}$ Ala2); 18.7 ($C^{β}$ Ala1); 18.7(5) ($C^{β}$ Ala5); 19.3 ($C^{β}$ Ala4); 22.9 (C^{Ac} N-terminal); 23.4 (C^{Ac} Gal); 26.0 (Me C-terminal); 48.3 ($C^{α}$ Ala1); 48.3(5) ($C^{α}$ Ala4); 48.7 ($C^{α}$ Ala2); 48.7(5) ($C^{α}$ Ala5); 49.7 (C^2 Gal); 57.2 ($C^{α}$ Thr3); 60.8 (C^6 Gal); 67.9 (C^3 Gal); 68.6 (C^4 Gal); 69.4 ($C^{β}$ Thr3); 71.3 (C^5 Gal); 93.1 (C^1 Gal); 169.1 (C(O) Thr3); 169.5 ($C(O)^{Ac}$ N-termina); 170.0 (C(O) Gal); 172.2 (C(O) Ala4); 172.7 (C(O) Ala5); 172.8 (C(O) Ala1); 173.2 (C(O) Ala2)
$C_{27}H_{47}N_7O_{12}$
M = 661.71 g/mol
Exact mass: 661.33
MS (ESI) = 662.3391 [M + H]$^+$

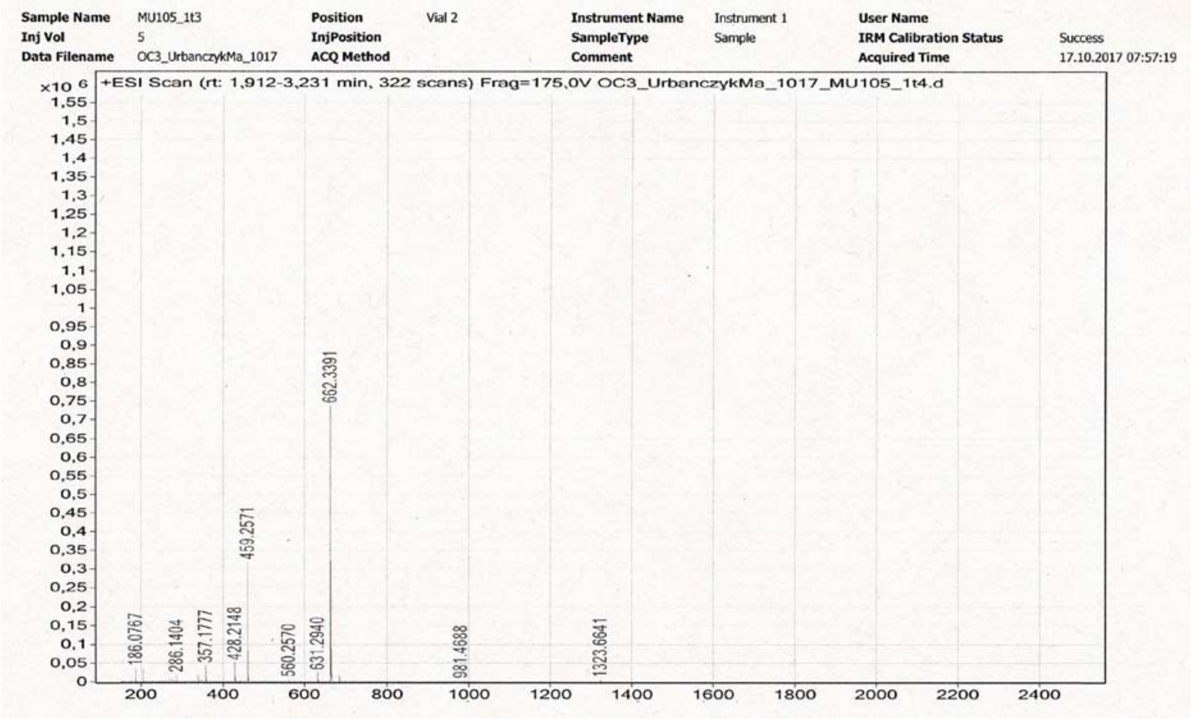

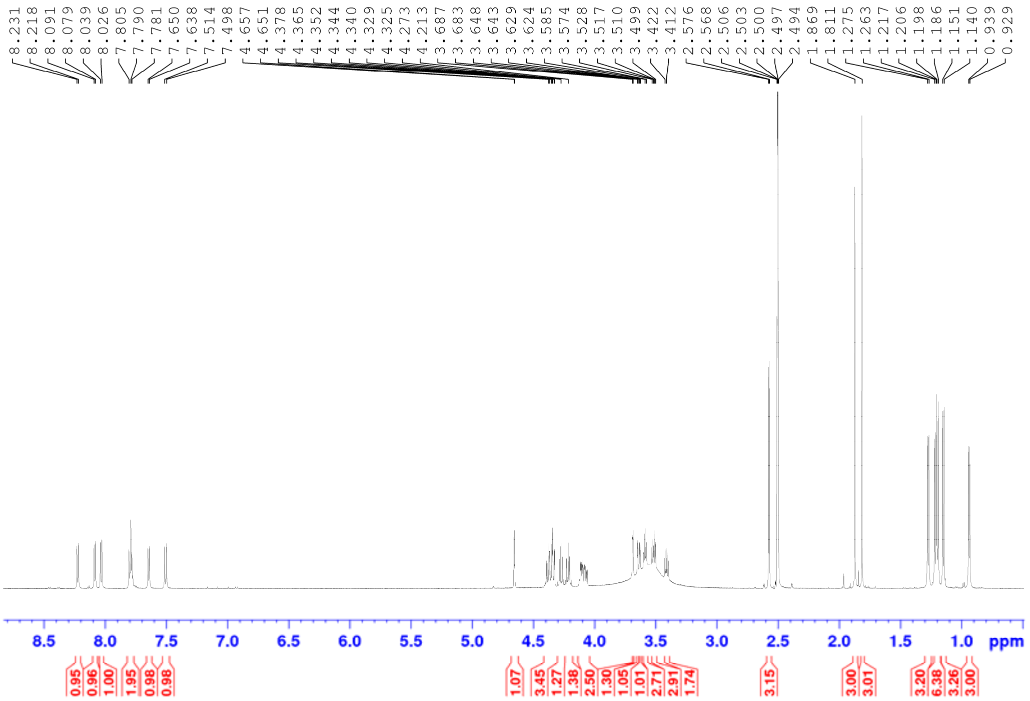
8.231 8.218 8.091 8.079 8.039 8.026 7.805 7.790 7.781 7.650 7.638 7.514 7.498 4.657 4.651 4.378 4.365 4.352 4.344 4.340 4.329 4.325 4.273 4.213 3.687 3.683 3.648 3.643 3.629 3.624 3.585 3.574 3.528 3.517 3.510 3.499 3.422 3.412 2.576 2.568 2.506 2.503 2.500 2.497 2.494 1.869 1.811 1.275 1.263 1.217 1.206 1.198 1.186 1.151 1.140 0.939 0.929
8.5 8.0 7.5 7.0 6.5 6.0 5.5 5.0 4.5 4.0 3.5 3.0 2.5 2.0 1.5 1.0 ppm
0.95 0.96 1.00 1.95 0.98 0.98 1.07 3.45 1.27 1.38 2.50 1.30 1.05 1.01 2.71 2.91 1.74 3.15 3.00 3.01 3.20 6.38 3.26 3.00

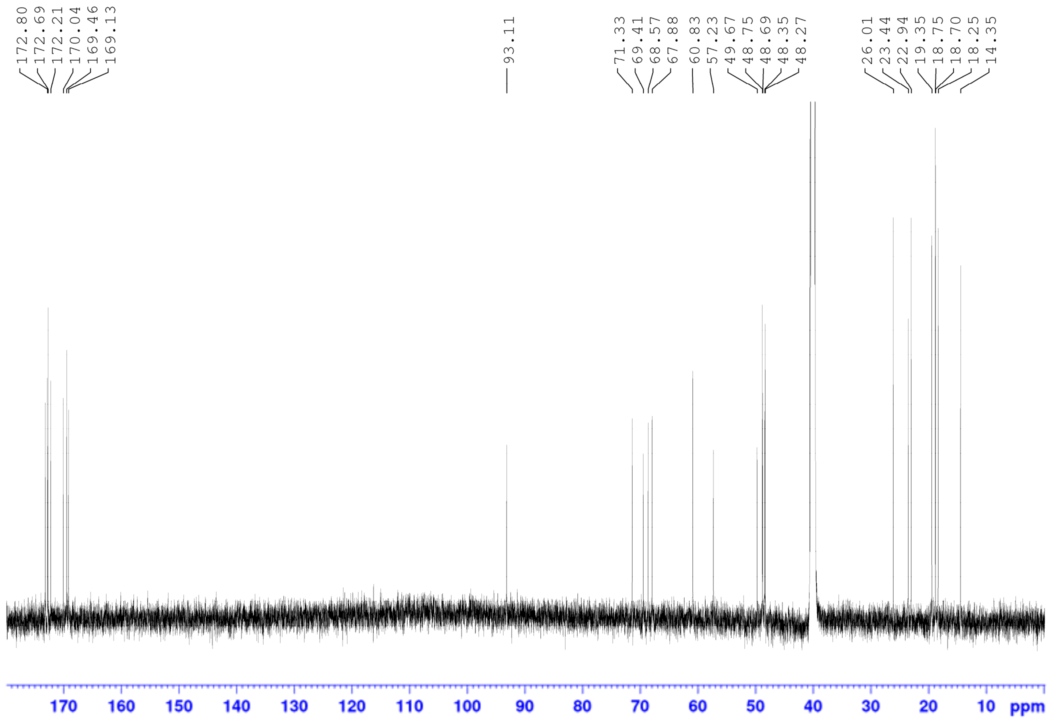
172.80 172.69 172.21 170.04 169.46 169.13 93.11 71.33 69.41 68.57 67.88 60.83 57.23 49.67 48.75 48.69 48.35 48.27 26.01 23.44 22.94 19.35 18.75 18.70 18.25 14.35
170 160 150 140 130 120 110 100 90 80 70 60 50 40 30 20 10 ppm

Table S3. Interatomic distances determined on the ROESY experiment for **1**.

Residue name	Residue number	Atom name	Residue name	Residue number	Atom name	Distance [Ang]
Ac	1	H	Ala	2	HA	3.59
Ac	1	H	Ala	2	HN	2.45
Ala	2	HA	Ala	2	HN	3.06
Ala	2	HA	Thr	3	HN	2.47
Ala	2	HB	Ala	2	HA	2.53
Ala	2	HB	Ala	2	HN	2.56

Table S4. Interatomic distances determined on the ROESY experiment for **2**.

Residue name	Residue number	Atom name	Residue name	Residue number	Atom name	Distance [Ang]
Ac	1	HA#	Ala	2	HN	2.44
Ala	2	HA	Ala	2	HN	3.10
Ala	2	HA	Thr	3	HN	2.37
Ala	2	HB	Ala	2	HA	2.42
Ala	2	HB	Ala	2	HN	2.61
Ala	2	HB	Thr	3	HN	2.01
Thr	3	HA	Ala	4	HN	2.69
Thr	3	HB	Thr	3	HA	2.72
Thr	3	HB	Thr	3	HN	3.21
Thr	3	HB	Thr/GalNAc	3	H1	2.72
Thr	3	HG	Ala	4	HN	2.53
Ala	3	HG	CNMe[a]	5	HN	3.17
Ala	4	HA	Ala	4	HN	2.27
Ala	4	HA	CNMe[a]	5	HN	2.23
Ala	4	HB	Thr	3	HA	2.79
Ala	4	HB	Thr	3	HN	2.93
Ala	4	HB	Ala	4	HA	2.66
Ala	4	HN	Thr	3	HN	2.39
CNMe[a]	5	HA	CNMe[a]	5	HN	3.19
CNMe[a]	5	HA	ThrGalNAc	3	H4	4.57
Thr/GalNAc	3	Me	Thr/GalNAc	3	HN2	2.49
Thr/GalNAc	3	H2	Thr/GalNAc	3	H1	2.25
Thr/GalNAc	3	H2	Thr/GalNAc	3	HN2	2.20
Thr/GalNAc	3	H3	Thr/GalNAc	3	H1	3.09
Thr/GalNAc	3	H3	Thr/GalNAc	3	HN2	2.57
Thr/GalNAc	3	H62	Thr/GalNAc	3	H1	3.08
Thr/GalNAc	3	HN2	Thr	3	HN	2.13

[a]CNMe – HN of C-terminal group

Table S5. Interatomic distances determined on the ROESY experiment for **3**.

Residue name	Residue number	Atom name	Residue name	Residue number	Atom name	Distance [Ang]
Ac	1	H	Ala	2	HN	3.51
Ac	1	H	Ala	5	HN	3.43
Ac	1	H	Thr	4	HN	3.06
Ala	2	HA	Ala	3	HN	2.33
Ala	2	HB	Ala	2	HN	2.54
Ala	3	HA	Ala	2	HN	2.68
Ala	3	HA	Ala	3	HN	2.84

Ala	3	HB	Ala	3	HN	2.39
Thr	4	HA	Thr	4	HN	2.08
Thr	4	HG	Thr	4	HA	2.58

Table S6. Interatomic distances determined on the ROESY experiment for **4**.

Residue name	Residue number	Atom name	Residue name	Residue number	Atom name	Distance [Ang]
Ala	2	HB	Ala	2	HA	2.05
Ala	2	HN	Ac	1	H	2.21
Ala	2	HN	Ala	2	HA	2.88
Ala	2	HN	Ala	2	HB	2.21
Ala	3	HB	Ala	3	HA	2.07
Ala	3	HN	Ala	2	HA	1.82
Ala	3	HN	Ala	3	HA	2.16
Ala	3	HN	Ala	3	HB	2.30
Thr/GalNAc	4	H1	Thr/GalNAc	4	H2	2.18
Thr/GalNAc	4	H1	Thr/GalNAc	4	H5	2.99
Thr/GalNAc	4	H1	Thr/GalNAc	4	HN2	2.51
Thr/GalNAc	4	H2	Thr/GalNAc	4	H3	2.26
Thr/GalNAc	4	H2	Thr/GalNAc	4	H4	2.71
Thr/GalNAc	4	H2	Thr/GalNAc	4	HN2	2.61
Thr/GalNAc	4	H3	Thr/GalNAc	4	H5	2.41
Thr/GalNAc	4	H3	Thr/GalNAc	4	HN2	2.03
Thr/GalNAc	4	H4	Thr/GalNAc	4	H5	2.10
Thr/GalNAc	4	H4	Thr/GalNAc	4	H61	2.33
Thr/GalNAc	4	H62	Thr/GalNAc	4	H5	2.41
Thr/GalNAc	4	H62	Thr/GalNAc	4	H61	1.78
Thr	4	HB	Thr/GalNAc	4	H5	2.29
Thr	4	HB	Thr	4	HA	2.01
Thr	4	HB	Thr	5	HN	2.46
Thr	4	HG2	Thr/GalNAc	4	H1	2.19
Thr	4	HG2	Thr	4	HA	2.41
Thr	4	HG2	Thr	4	HB	2.07
Thr	4	HN	Ala	3	HA	1.80
Thr	4	HN	Ala	3	HB	2.74
Thr	4	HN	Thr	4	HB	2.63
Thr	4	HN	Thr	4	HG2	2.54
Thr/GalNAc	4	HN2	Thr/GalNAc	4	H1	2.54
Thr/GalNAc	4	HN2	Thr/GalNAc	4	H2	2.55
Thr/GalNAc	4	HN2	Thr	4	HG2	3.21
Thr/GalNAc	4	HN2	Thr/GalNAc	4	Me	2.12
Thr/GalNAc	4	HN2	Thr	4	HN	2.38
Ala	5	HB	Thr/GalNAc	4	H3	2.30
Ala	5	HB	Thr/GalNAc	4	H4	3.08
Ala	5	HB	Thr/GalNAc	4	H5	2.86
Ala	5	HB	Ala	5	HA	2.05
Ala	5	HN	Ala	3	HB	2.92
Ala	5	HN	Thr/GalNAc	4	H3	2.18
Ala	5	HN	Thr/GalNAc	4	H5	2.98
Ala	5	HN	Thr	4	HB	2.45
Ala	5	HN	Thr	4	HN	2.03
Ala	5	HN	Ala	5	HB	2.35
Ala	6	HB	Ala	6	HA	1.98
Ala	6	HN	Ala	5	HA	1.86

Ala	6	HN	Ala	6	HA	2.14
Ala	6	HN	Ala	6	HB	2.25
CNMe[a]	7	HN	Ala	6	HB	2.82

[a]CNMe – HN of C-terminal group
